# Evaluation of CoFe_2_O_4_-L-Au (L: Citrate, Glycine) as Superparamagnetic–Plasmonic Nanocomposites for Enhanced Cytotoxic Activity Towards Oncogenic (A549) Cells

**DOI:** 10.3390/ijms26167732

**Published:** 2025-08-10

**Authors:** Alberto Lozano-López, Mario E. Cano-González, J. Ventura-Juárez, Martín H. Muñoz-Ortega, Israel Betancourt, Juan Antonio Zapien, Iliana E. Medina-Ramirez

**Affiliations:** 1 Departamento de Química, Centro de Ciencias Básicas, Universidad Autónoma de Aguascalientes, Av. Universidad #940, Aguascalientes C.P. 20100, Aguascalientes, Mexico; albertolozano93@gmail.com (A.L.-L.); martinenol@hotmail.com (M.H.M.-O.); 2 Centro Universitario de la Ciénega, Universidad de Guadalajara, Av. Universidad #1115, Col. Linda Vista, Ocotlán C.P 47810, Jalisco, Mexico; mario.cano@academicos.udg.mx; 3 Departamento de Morfología, Centro de Ciencias Básicas, Universidad Autónoma de Aguascalientes, Av. Universidad #940, Aguascalientes C.P. 20100, Aguascalientes, Mexico; javier.ventura@edu.uaa.mx; 4 Instituto de Investigaciones en Materiales, Universidad Nacional Autónoma de México, Mexico City C.P. 04510, Mexico; israelb@unam.mx; 5 Department of Materials Science and Engineering, City University of Hong Kong, Hong Kong SAR, China

**Keywords:** cancer therapy, CoFe_2_O_4_, CoFe_2_O_4_-Au, nano–bio interactions, photodynamic therapy, cytotoxicity

## Abstract

We investigated the influence of gold deposition on the magnetic behavior, biocompatibility, and bioactivity of CoFe_2_O_4_ (MCF) nanomaterials (NMs) functionalized with sodium citrate (Cit) or glycine (Gly). The resulting multifunctional plasmonic nanostructured materials (MCF-Au-L, where L is Cit, Gly) exhibit superparamagnetic behavior with magnetic saturation of 59 emu/g, 55 emu/g, and 60 emu/g, and blocking temperatures of 259 K, 311 K, and 322 K for pristine MCF, MCF-Au-Gly, and MCF-Au-Cit, respectively. The MCF NMs exhibit a small uniform size (with a mean size of 7.1 nm) and an atomic ratio of Fe:Co (2:1). The gold nanoparticles (AuNPs) show high heterogeneity as determined by high-resolution transmission electron microscopy (HR-TEM) and energy-dispersive X-ray spectroscopy (EDX). The UV-Vis spectroscopy of the composites reveals two localized surface plasmons (LSPs) at 530 nm and 705 nm, while Fourier Transformed-Infrared spectroscopy (FTIR) and thermogravimetric analysis (TGA) confirm the presence of Cit and Gly on their surface. Subsequent biocompatibility tests confirm that MCF-Au-L NMs do not exert hemolytic activity (hemolysis < 5%). In addition, the CCK-8 viability assay tests indicate the higher sensitivity of cancerous cells (A549) to the photoactivity of MCF-Au compared to healthy Detroit 548 (D548) cell lines. We use advanced microscopy techniques, namely atomic force, fluorescence, and holotomography microscopies (AFM, FM, and HTM, respectively) to provide further insights into the nature of the observed photoactivity of MCF-Au-L NMs. In addition, in situ radiation, using a modified HTM microscope with an IR laser accessory, demonstrates the photoactivity of the MCF-Au NMs and their suitability for destroying cancerous cells through photodynamic therapy. The combined imaging capabilities demonstrate clear morphological changes, NMs internalization, and oxidative damage. Our results confirm that the fabricated multifunctional NMs exhibit high stability in aqueous solution, chemical solidity, superparamagnetic behavior, and effective IR responses, making them promising precursors for hybrid cancer therapy.

## 1. Introduction

Nowadays, the most common ways to treat cancer are chemotherapy and radiotherapy. Those treatments, however, have secondary effects due to the inability to distinguish between healthy and dysplastic cells and new technologies are necessary to fight cancer while avoiding or minimizing secondary effects [1,2]. Magnetic hyperthermia therapy (MHT) is a promising alternative to treat cancerous cells and clinical trials have demonstrated its effectiveness as an adjuvant for chemotherapy and radiotherapy treatments in reducing the size of dysplastic tissues [3]. Using magnetic nanostructured materials (MNMs) results in enhanced and specific heat transfer. In addition, the selectivity of MHT increases by conjugating the cell-targeting ligands to the MNM surface [4]. Furthermore, the surface of MNMs can serve as a platform to conjugate multiple tumor targeting and therapeutic moieties (biotherapeutics, chemotherapeutics, photo-functional moiety) [5], leading to hybrid therapies with higher efficiencies to treat solid tumors.

Previous studies (2020) from our research group established the heat transfer capability and biocompatibility of MCF NMs with human cell lines (Hep-G2) and showed that these materials were new promising alternatives for MHT [1]. However, due to the complex nature of cancerous tissue, it is necessary to expand the cytotoxic activity of these materials to achieve higher malignant cell destruction rates with therapy. In fact, recent studies have shown that hybrid materials can promote enhanced cytotoxic activity when their multifunctional bioactivity is capable of targeting cancer cells in multiple ways. For example, magnetite NMs coupled to cancer chemotherapeutic drugs have been shown to exert enhanced anticancer activity [6].

Furthermore, MHT can enhance immune therapies by different mechanisms. In fact, recent studies have shown that hybrid materials can promote enhanced cytotoxic activity when their multifunctional bioactivity is capable of targeting cancer cells in multiple ways including, for example, (i) coupling to cancer chemotherapeutic drugs for enhanced anticancer activity [6]; (ii) increasing leukocytes traffic by releasing high concentrations of chemoattractant specifically for those cells [7]; (iii) strengthening immunotherapy with negligible toxicity [8]; (iv) targeting tumor tissue and delivering heating to kill cancerous cells; and (v) increasing tumor immunogenicity and tumor permeability, thereby improving immune cell infiltration and making solid tumors more responsive to immunotherapy [9]. Previous research has described how MHT induces physiological changes and apoptosis in neoplastic cells through various mechanisms. The most relevant factors are the induction of DNA damage through reactive oxygen species (ROS) formation, cell cycle arrest, and protein denaturation [10].

In addition, numerous studies indicate that thermal ablation caused by photodynamic therapy (PDT) can reduce prostatic cancer tumors [11]. The use of plasmonic nanoparticles represents another promising alternative to the targeted and localized destruction of cancerous cells [12]. Plasmonic materials can sustain Localized Surface Plasmon Resonances (LSPRs) that occur when an electromagnetic field interacts with a metal particle, raising the collective oscillation of electrons in resonance with the incident field [13]. LSRP results in highly enhanced electromagnetic fields with application for sensing [14], the localized temperature control of use in thermal engineering [15], and the creation of metastable, hot electrons [8]. Hot electrons remain in this metastable state until the plasmon decays to the lowest energy state, transferring the energy to the closest molecule, which is often oxygen and has a triplet character (^3^O_2_) in its ground state. This energy transfer is the basis for photodynamic therapy (PDT) based on the formation of ROS (^1^O_2_, –OH, H_2_O_2_, and O_2_^−^) capable of oxidizing cellular components, compromising cellular integrity, and leading to cellular death. On the other hand, thermal effects in LSPR lead to photothermal therapy (PTT), which is used to treat cancer tumors by means of coagulative or hyperthermic thermal ablation [11,16].

Among many plasmonic materials, AuNPs arouse great attention for their potential therapeutic effects (drug delivery, gene therapy, counter-cancer agents) against cancerous cells. For example, stimuli-responsive AuNPs have been used to develop efficient and precise techniques for cancer theragnostic [17]. To date, several reports on the synergic use of MHT and plasmonic-based PTT and PDT have highlighted the enormous potential of this hybrid approach [18,19]. However, several key challenges remain and have been recently discussed including poor photothermal conversion efficiency (PCE), heat resistance, limited accumulation in tumor, cytotoxicity, and others [20]. Several of these issues, including the aggregation of NPs and its effects on their optical and thermal properties, affecting PCE as well as biocompatibility, can be traced to the physicochemical surface properties of the NMs which can be efficiently modified by surface functionalization.

In this work, we present MNMs conformed by MCF functionalized with citrate (Cit) or glycine (Gly) and with AuNPs deposited into their surface (MCF-Au-Cit and MCF-Au-Gly). The magnetic core (CoFe_2_O_4_) has tuned properties to exert a high heat transfer for MHT, whereas the AuNPs are responsive to IR activation for PDT (as complementary treatments against cancer). In addition, toxicology assays (hemolytic activity and CCK8 assay) support the biocompatibility of the materials, indicating a higher cytotoxicity of the composites for A549 (lung alveolar cancer) in comparison to Detroit 548 (skin fibroblasts), demonstrating the affinity of the materials for cancerous cells. Supporting these findings, analysis of the interaction of NMs with cells using advanced microscopy techniques (AFM, HTM, and fluorescence microscopy) reveals the morphological changes. It supports the entry of the composites into cancerous cells, followed by ROS production upon the light activation of the materials. To our knowledge, this is the first study using in situ IR radiation to monitor the effects of PDT in cancerous cells in real time.

## 2. Results and Discussion

### 2.1. Synthesis of MCF, MCF-AuCit, and MCF-AuGly

In previous reports, the advantages of using a microwave-assisted solvothermal route and benzyl alcohol to synthesize nanostructured metal oxides were discussed [1,21,22]. Due to the control that this synthesis technique provides, MCF exhibits a small nanometric size (diameter) with narrow dispersion (7 nm ± 1.3 nm) (Figure S1), purity, and crystallinity (corroborated by HRTEM and XRD analyses). In addition, this synthetic approach has a high repeatability rate and yields of reaction (85 ± 6%).

Furthermore, in this study, citrate and glycine serve as functionalization agents to enhance the colloidal stability and biocompatibility of MCF. FTIR and TGA analyses showed that the functionalization route was adequate to deposit Cit and Gly on the MCF surface and to improve colloidal stability (Figure S2) [1]. Figure S2 shows the colloidal stability of MCF and MCF-Au (1 mg/mL) in Arabic gum (3% *w*/*v*) at different time intervals (0, 16, and 24 h). Naked magnetic cores exhibit moderate stability (Figure S2(A1–A3)) in Arabic gum over the time-lapse (24 h) of evaluation. The colloidal stability of MCF improves after citrate functionalization (Figure S2(D1–D3)); however, glycine-functionalized composites (MCF-AuGly) exhibit enhanced colloidal stability (Figure S2(C1–C3)).

The properties of bare magnetic nanomaterials (MNMs) are far from ideal for biomedical applications, particularly due to their aggregation in aqueous media and poor chemical stability (oxidation), which results in a low and irreproducible magnetic response [23]. The functionalization of MNMs leads to an improvement in their physicochemical properties, favoring their biomedical applications. The conditions for the functionalization of the MNMs to suit their biomedical applications aim (a) to improve colloidal stability, (b) to increase their water stability, (c) to ensure better and homogeneous magnetic performance, (d) to protect and stabilize their surface, (e) to improve their biocompatibility, (f) and to supply bare functional groups to attach biologically active substances for nano–bio applications [24].

Previous studies from our research group draw evidence of the effectiveness of citrate and glycine in enhancing the colloidal and chemical stability of MNMs (Fe_3_O_4_, CoFe_2_O_4_, CuFe_2_O_4_, ZnO-CuFe_2_O_4_) [1,25]. Citrate is a biomolecule with redox properties, biocompatibility, and a chemical composition that provides electrostatic and steric stabilization to the MNMs to achieve an equilibrium between attractive and repulsive forces, leading to colloidal and chemical stability. Citrate also supplies bare functional groups that interact with biologically active substances (in this study with gold) to render multifunctional MNMs [23,24].

Molecules containing carboxylic acid groups, such as sodium citrate, are used in the synthesis and functionalization of metal oxide (TiO_2_, Fe_3_O_4_, ZnO) NPs [26]. The oxygen anions of the carboxylate groups are hard bases; thus, according to the hard–soft acid–base theory, they coordinate effectively with hard acids, such as metal cations with high oxidation numbers and small ionic radii. In addition, the citrate-functionalized NMs exhibit a negative zeta potential due to the anionic carboxylate groups of the deprotonated ligands. The electrostatic repulsion from the negatively charged surface favors colloidal stability [20].

Glycine (Gly) is an amino acid that possesses both carboxylic (a hard Lewis base, mainly negatively charged at physiological pH values) and amino (a borderline Lewis base, mainly positively charged at physiological pH values) groups, increasing the possibilities for chemical interactions with biologically active substances. The carboxylic group of Gly can interact with hard Lewis’s acids. In contrast, the amine group (situated on the borderline between a hard and a soft Lewis base) interacts with soft Lewis’s acids (i.e., gold). In addition, these functional groups can be charged (positively or negatively) depending on the pH of the media, contributing to the electrostatic repulsions and stability of the colloidal suspension [27].

**Figure 1:**
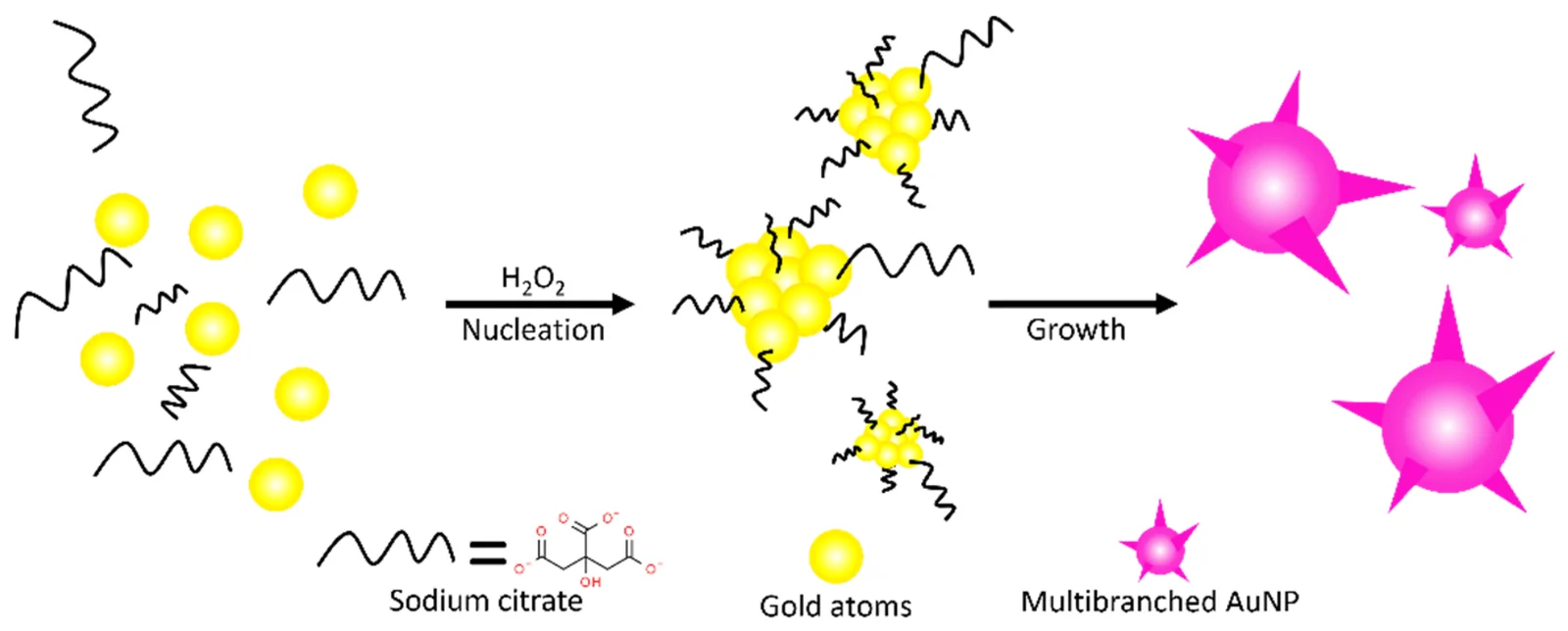
Representation of multibranched AuNP synthesis mechanism with Cit presence and H_2_O_2_ as reducing agent. As Au seeds grow (due to the H_2_O_2_ presence), Cit binds to Au seeds with no preferential site, generating moieties and inducing formation of branches.

Because of their biomedical applications, it is necessary to optimize green routes to produce branched Au NPs. In addition to their non-toxic nature, gold nanostructures must be responsive to infrared excitation to avoid the problems associated with UV or visible light activation (limited depth penetration in tissues and low therapeutic efficiency for deep lesions). In this study, a green approach was followed to produce branched Au NPs, using citrate as a capping agent and hydrogen peroxide to reduce Au^3+^ ions to metallic particles. The formation of multibranched AuNPs is mediated by the molar ratio between Cit and Au. Due to the Cit affinity to Au [28], Cit becomes a capping agent that couples on the Au seed’s surface with no preferential plane, generating anisotropic growth, creating the branches, and broadening the size dispersion (Equation (1) and Figure 1) [29,30]. The colloidal and optical properties of the produced materials indicate that the reaction proceeded as expected, forming AuNPs that are responsive in the infrared region (Figure S3).

(1) $${Au}^{3+}+H_{2}O_{2}\to Au+{2H}^{+}+O_{2}\;\;\;$$

Several strategies exist to produce branched Au NPs. However, some involve toxic surfactants (CTAB), limiting the biocompatibility of the AuNPs. In this study, anisotropic Au NMs are produced using a green and fast approach. As illustrated in Figure 1, the Cit-Au molar ratio becomes a key parameter for the formation of multibranched structures. As the Cit concentration increases (Figure S3), the gold seeds increase the capping of the Cit shell, preventing the formation of branches and causing the second LSPR to disappear. As prepared, the Au colloid is the starting material used to produce MCF-Au composites. Previous studies discussed the low stability of colloidal branched Au nanostructures at room temperature. In these conditions, the branched Au nanostructures tend to rearrange into thermodynamically favorable spherical NPs at room temperature [31]. To avoid structural changes in the prepared AuNPs, freshly prepared suspensions are used for gold deposition; however, further studies will explore the use of biopolymers to increase the stability of colloidal branched nanostructures. The present investigation involves low gold concentrations and the avoidance of polymer protection to prevent the modification of the magnetic properties of MCF cores.

Despite the advances in drug design and delivery, cancer treatment is still challenging. Nanotechnology offers promising alternatives for the design of targeted and effective cancer therapies. Although numerous efforts are currently directed to the design and fabrication of nanomaterials with optimal properties for cancer treatment, there are few examples of the translation of these NMs to clinical application [16,32]. A recent study remarked on the challenges and future perspectives on the development of NMs for cancer treatment (photothermal). Among these challenges appear to be problems associated with the stability and production of the NMs, as well as their biocompatibility, mode of action, and therapeutic efficacy [16].

In this study, we employ a microwave solvothermal route that yields magnetic materials with tunable properties. As previously discussed, the kinetics of this synthetic approach ease the fabrication of magnetic NMs with varied properties (i.e., composition, Fe_3_O_4_, CoFe_2_O4, MnFe_2_O_4_, CuFe_2_O_4_, ZnFe_2_O_4_). In addition, the materials can be easily functionalized without changing their magnetic properties; this functionalization directs the incorporation of additional moieties on the surface of the magnetic core. Within this investigation, plasmonic nanoparticles are deposited on the surface of the magnetic core to fabricate hybrid composites sensitive to magnetic field and IR radiation stimuli. Plasmonic particles are produced using a green approach, rendering biocompatible and bioactive materials. The versatility of the composite design allows the further modification of their surface, rendering multifunctional materials excitable by different means; thus, diverse therapeutic mechanisms are used to destroy oncogenic cells.

### 2.2. Characterization of Materials

#### 2.2.1. HRTEM/EDX Analysis

**Figure 2:**
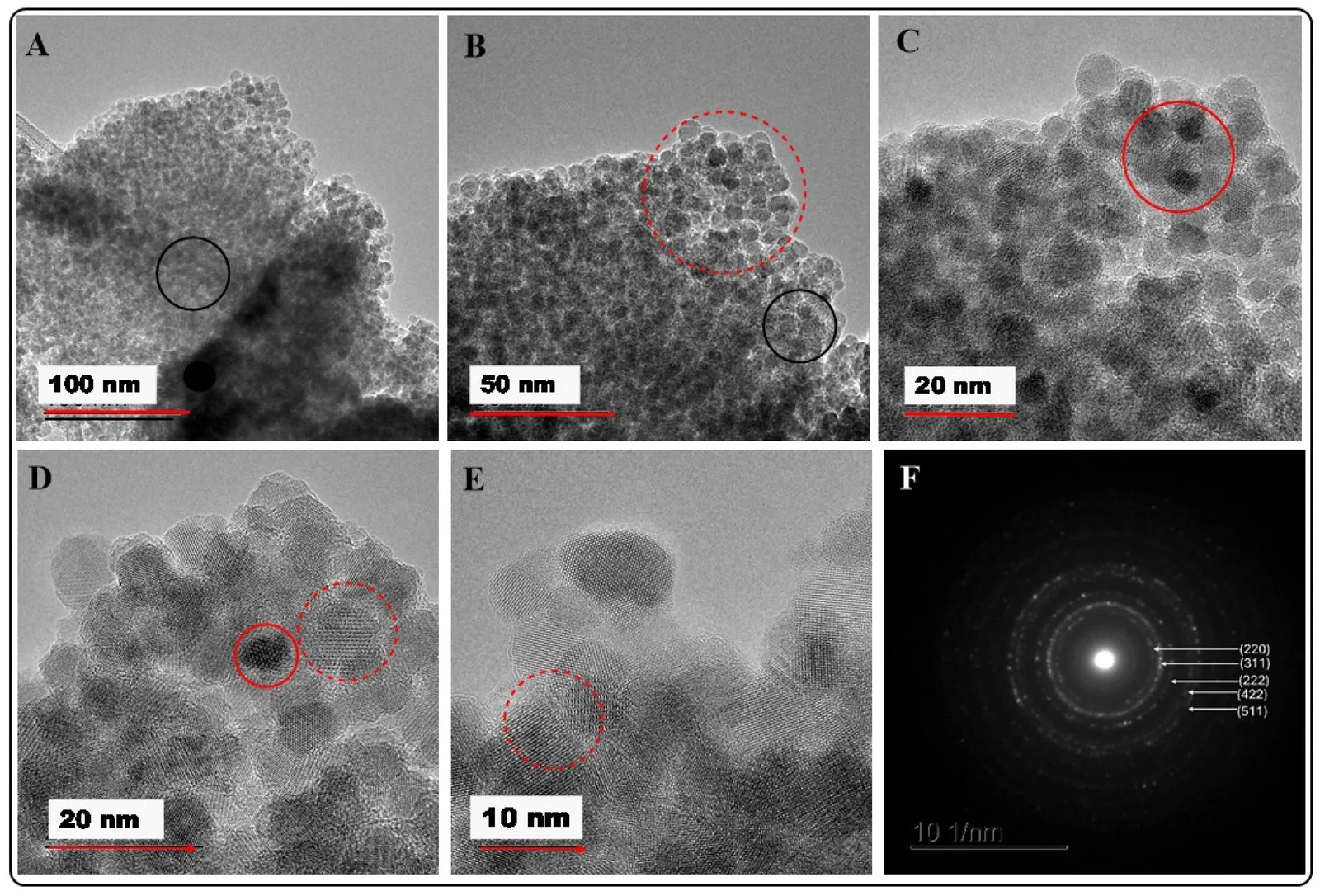
(**A**,**B**) Low-magnification TEM micrographs of MCF-AuGly, displaying aggregates of the MCF-Au NMs. Higher contrast indicates aggregation or Au deposition (**C**) CoFe_2_O_4_ reveals high monocrystalline and single orientation. (**D**,**E**) TEM micrographs of CoFe_2_O_4_ denoting their size and crystallinity. (**F**) Due to low Au in the region, SAED does not present significant signs of Au reflections. Black circles (CoFe_2_O_4_ agglomerates); red solid circles (zones of higher Au deposition); red dashed circles (High crystallinity of CoFe_2_O_4_).

**Figure 3:**
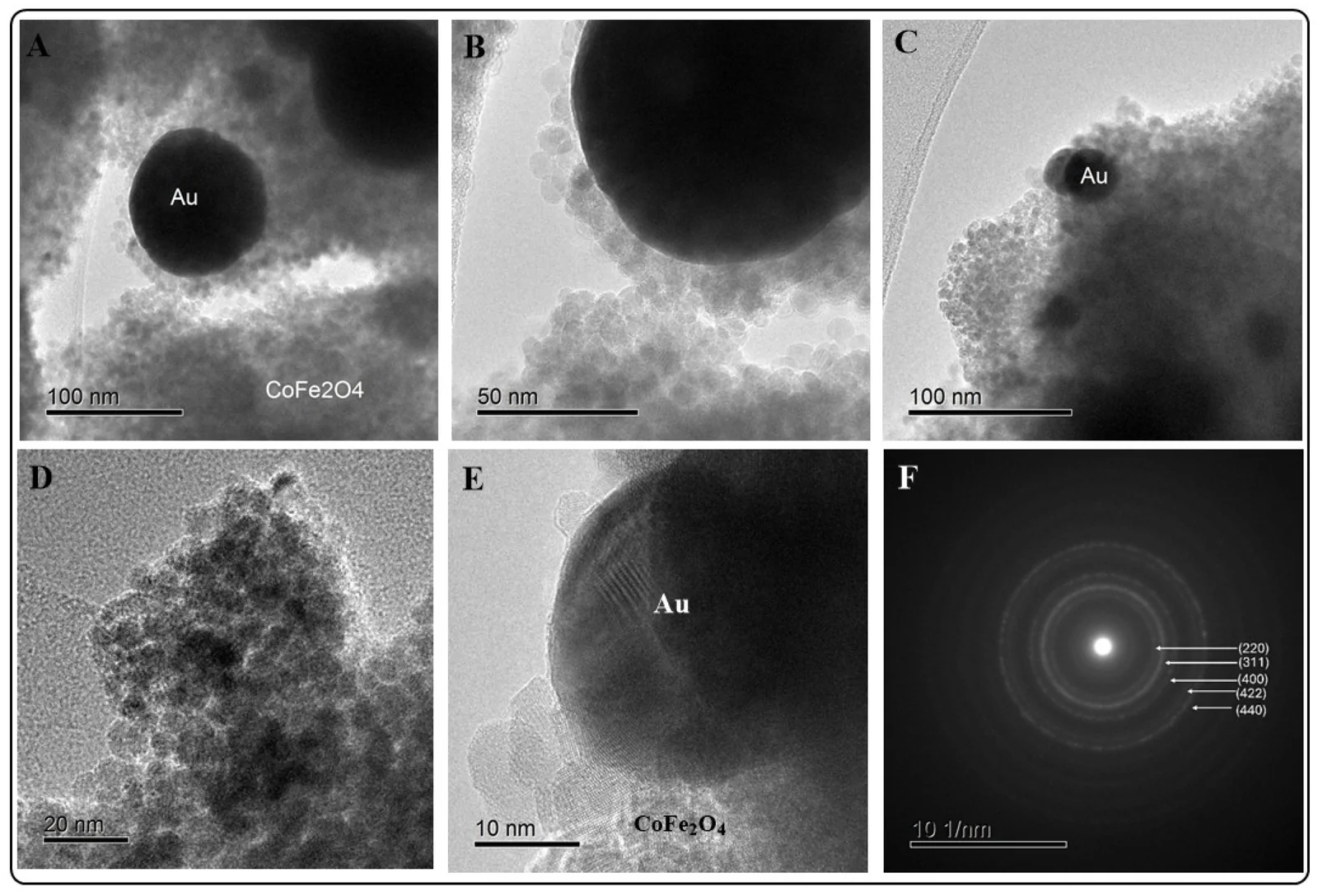
TEM and HRTEM images of MCF-AuCit at different magnifications. (**A**–**C**) TEM images of MCF-AuCit showing the presence of Au particles surrounded by MCF. Darker contrast corresponds to AuNPs (with a wide particle size range; 60 ± 36 nm), while lighter contrast corresponds to MCF; the contrast is due to differences in electron density. (**D**–**F**) HRTEM images of MCF-Au Cit. The images illustrate the small and uniform size of MCF (**D**). The reduced size and crystallinity of MCF are illustrated in (**E**). As a result of the low Au concentration in the scanned area, the corresponding SAED (**F**) does not present significant signs of Au reflections. (**D**–**F**) HRTEM images of MCF-Au Cit.

The HRTEM/EDX analysis of the MCF-Au reveals the uniform size, crystallinity, and purity of MCF-Au composites (Figure 2, Figure 3 and Figure S4). As can be seen, MCF has a spherical morphology, uniform size, and a tendency to form agglomerates (dashed red circle, Figure 2B). The EDX analysis (Figure S4 and Table S1) corroborates the formation of the magnetic core (CoFe_2_O_4_) with a small amount of gold on their surfaces (MCF-AuCit and MCF-AuGly).

The HRTEM analysis of MCF-AuGly reveals zones with higher opacity that may be present due to the higher Au deposition (plain red circle, Figure 2C,D). It also reveals their spherical morphology (Figure 2), homogeneous size distribution (Figure S1A), a tendency to form agglomerates (plain black circles, Figure 2A,B), and high crystallinity (dashed red circle, Figure 2D,E). In addition, EDX analysis supports the existence of Au (Figure S4A) in the MCF-Au. The Selected Area Electron Diffraction pattern (SAED) (Figure 2F) data demonstrate high crystallinity due to the well-defined reflections of the planes; however, these reflections only correspond to MCF (JCPDS 22-1086), consistent with XRD analysis, due to the low presence of gold in the samples.

**Figure 4:**
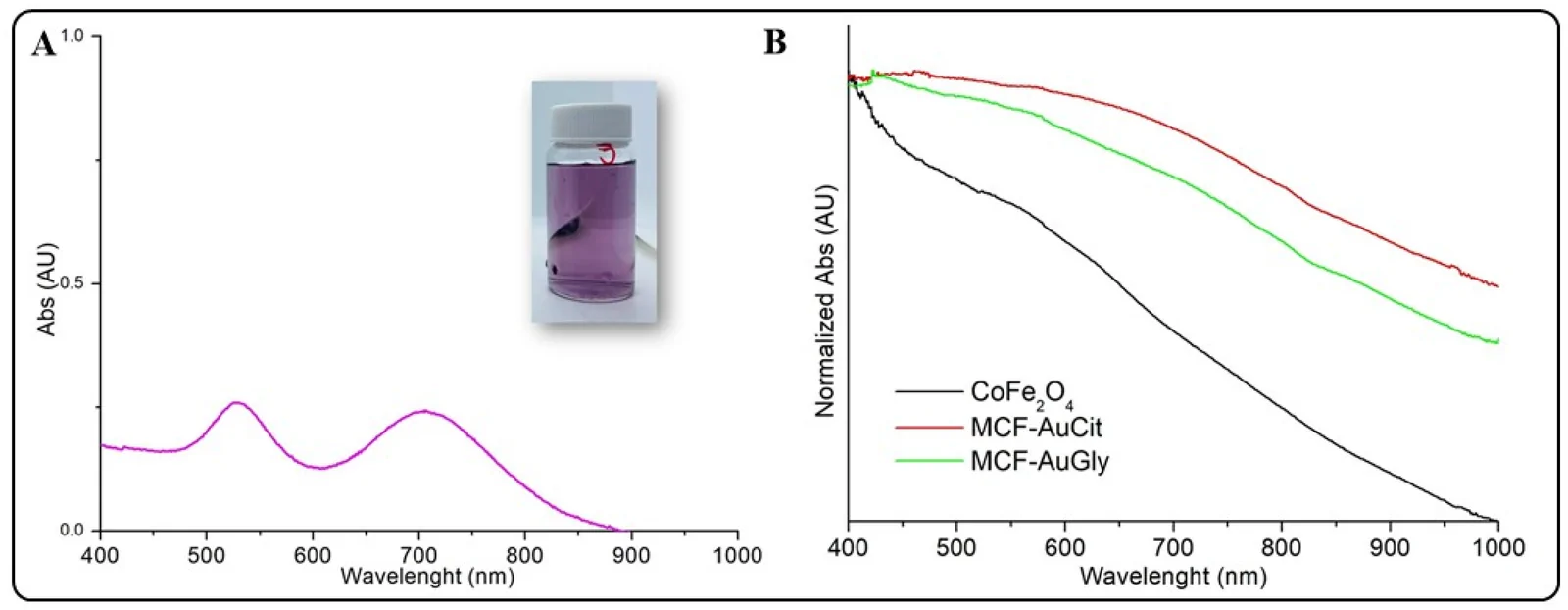
UV-Vis spectra of (**A**) pristine AuNPs (insert of the colloidal AuNPs). Due to the multibranched morphology, AuNPs exhibit two absorbances, with the first (~530 nm) related to the core, and the second (~720 nm) corresponding to anisotropic growth. (**B**) There is an increase in the absorption of the composites (MCF-AuCit, MCF-AuGly) due to the presence of Au NPs on the surface of the NMs.

In the case of MCF-AuCit (Figure 3), there is a clear contrast between AuNP and CoFe_2_O_4_ (Figure 3A). In addition, EDX analysis (Figure S4B) confirms the presence of Au in the MCF-AuCit. AuNPs present a wide particle size range (60 ± 36 nm, Figure S1), consistent with UV-Vis spectroscopy (Figure 4). Also, in both materials, the SAED (Inserts in Figure 2F and Figure 3F) only shows signs of CoFe_2_O_4_ reflections, which is consistent with the XRD analysis, due to the low presence of gold in the samples.

#### 2.2.2. UV-Vis Spectrophotometry

To corroborate the formation of multibranched AuNP, UV-Vis spectrophotometry analysis was realized, expecting to observe two LSPRs due to the formation of anisotropic AuNPs. These phenomena occur in different regions of the electromagnetic spectrum. This is determined by the size and shape of the nanomaterials [33]. The UV-Vis spectra (Figure 4A) reveal two broad peaks in the visible and NIR regions attributed to the LSPR (527 nm and 705 nm). The first band is in the visible region due to the size of the AuNP core, and the second band is broad and red-shifted, indicating anisotropic growth (branch formation).

Compared with the CTAB synthesis of Au nanorods [34,35], Cit does not bind to some preferential plane in the Au surface of the seeds and therefore generates anisotropic growth and random length in the size of the branches. In addition, Figure 4B shows the increased absorbance in the visible light region of MCF-Au composites in contrast with pristine CoFe_2_O_4_. Compared with Saikova S. et al. (2021) [36], our results suggest that the NMs under study have a high absorption rate. In the following sections, our results demonstrate the activity (cytotoxicity and ROS analyses) of the NMs upon IR light activation [33].

#### 2.2.3. XRD Analysis

The crystalline structure of the materials was confirmed via XRD spectra (Figure S5). The diffraction peaks are clear and broad, showing a well-matched profile with the spinel structure; they are ubicated at 30°, 35.5°, 43°, 53°, 57°, and 62° 2*θ* degrees, which correspond to the (220), (311), (400), (422), (511) and (440) planes, respectively (JCPDS 22-1086). Using Scherrer’s equation and the plane with the most intense reflection (311), the crystallite size was calculated at 6.3 nm, consistent with HRTEM analysis. The same planes were identified for MCF-AuGly and MCF-AuCit, which corresponds to CoFe_2_O_4_; however, Au nanoparticles do not reveal any reflection, due to the low quantity present (below the detection limit of 4% of the XRD technique).

#### 2.2.4. Analysis of Magnetic Properties

**Figure 5:**
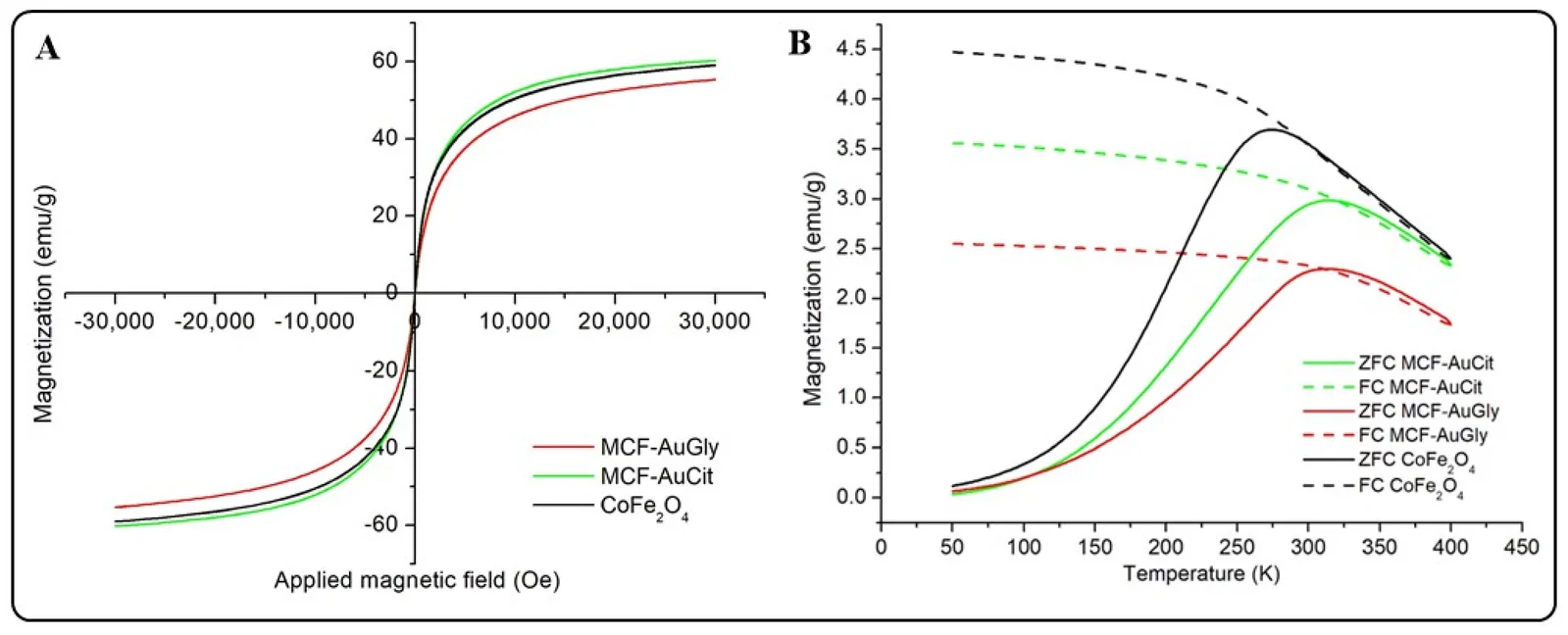
(**A**) Magnetic hysteresis loop of MCF-Au, (**B**) ZFC/FC hysteresis magnetization of the MCF-Au.

The magnetic response of the materials was analyzed using the VSM technique. In the magnetization versus applied magnetic field analysis (Figure 5A), the shape of the curves and the absence of magnetic hysteresis indicate that all materials have superparamagnetic behavior. The magnetic saturation (Ms) was found to be 59 emu/g, 55 emu/g, and 60 emu/g for MCF, MCF-AuGly, and MCF-AuCit, respectively; these high values may be attributed to the small size of the MCF-Au (Figure 5B). Gerina M. et al. (2023) [37] demonstrated the intrinsic relationship between the Ms and the particle size; due to the small number of atoms that compose a single MCF nanoparticle, the surface spin disorder has a bigger influence over the material’s magnetic properties, making them more susceptible to the magnetic fields. These results demonstrate that after functionalization and the addition of AuNP, the superparamagnetic behavior in all materials remains unchanged. Therefore, the construction of the Au composites does not significantly modify the magnetic properties of MCF. In addition, the Langevin model was applied to the MCF-Au curve to determine the magnetic moment per particle (*µ*). As is shown in Figure S6, the Langevin equation is fitted with good correlation (R^2^ = 0.99268) when *µ* = 5582 µB, which is consistent with the reported value of Medina et al. (2020) [1]. The difference between the Langevin fit and the experimental data may be attributed to the surface spin disorder in the nanoparticles and surface anisotropy [38].

Pristine MCF has a blocking temperature (Bt) of 297 °K, which is increased by the addition of AuNP, thereby expanding the range of the response in each material [36] (311 °K for MMCF-AuGly and 322 °K for MMCF-AuCit). This increase in Bt makes all MCF-Au NMs more suitable for MHT due to their response to magnetic fields at temperatures above 310 °K in biomedical applications [39].

#### 2.2.5. FTIR Spectroscopy

To confirm the addition of the functionalization molecules (Cit, Gly) to the surface of MCF, FTIR analysis was performed on the functionalized materials (MCF-Gly and MCF-Cit) in the range from 4000 cm^−1^ to 400 cm^−1^. In Figure S7A, a typical spectrum of CoFe_2_O_4_ is exhibited with well-defined peaks at 526 cm^−1^, 1081 cm^−1^, and 1196 cm^−1^, which are identified as Fe(III)-O^2−^ tetrahedral group complex stretching vibrations corresponding to the spinel skeleton of the structure. The peaks at 909 cm^−1^ and 1019 cm^−1^ are attributed to the characteristic Fe-Co bonds of the ferrite spinel structure, and the peak at 694 cm^−1^ is related to the Co(II)-O^2−^ octahedral group complex vibration [40,41]. The peaks located at 1396 cm^−1^ and 1541 cm^−1^ are assigned to the –OH of residual Fe–OH groups [42].

Figure S7B shows the analysis after functionalization with Gly. In the 3500 cm^−1^ to 2500 cm^−1^ region, a wide band is present because of the deformation vibration in the –OH and –NH_2_ groups due to the influence of hydrogen bonds [40]. This phenomenon is also present in the –NH_3_^+^ band vibration of the deformation peak in 1573 cm^−1^ and may be attributed to the zwitterionic nature of the amino acid [43]. Therefore, the presence of Gly is corroborated because of the bands of –OH and –NH_2_ corresponding to the amino acid [44]. FTIR analysis for MCF-Cit is depicted in Figure S7C. The peaks at 1391 cm^−1^, 1556 cm^−1^, and 2360 cm^−1^ are related to the vibration of the carboxylic groups from the citrate molecule. A thick and clear peak with a shoulder located at 1556 cm^−1^ is attributed to the –OH and C=O deformation vibrations, corroborating the presence of citrate in the surface of MCF NMs [40].

#### 2.2.6. TGA Analyses

TGA was performed as a complementary technique to quantify the organic mass present in the MCF-Au NMs. Based on the results (Figure S8A,B), the amount of organic mass for 10 mg of each sample is calculated, observing the following results: 1.8 mg Cit/10 mg MCF-Cit, 1.7 mg Cit/10 mg MCF-AuCit, 1.6 mg Gly/10 mg MCF-Gly, and 1.5 mg Gly/10 mg MCF-AuGly. This reveals that Cit and Gly have a high affinity for the MCF surface; even after the synthesis of the MCF-Au NMs, these organic molecules remain on the material due to the electrostatic attraction between them. Hence, the proposed methodology for the MCF-Au is appropriate for synthesizing MCF-Au with a functionalized surface.

Based on Cholico F. et al. (2022) [45], once the mass loss is measured, these results can be used to calculate the shell thickness of the coating material (Δ) using the mass fractions of the core (η_c_) and shell (η_s_) derived from the TGA (2).

(2) $$∆=\frac{\sigma_{c}}{2}\left({\left[\frac{\eta_{c}\rho_{s}}{\eta_{c}\rho_{s}+\eta_{s}\rho_{c}}\right]}^{\frac{-1}{3}}-1\right)\;\;\;$$

where σ_c_ is the nanoparticle diameter, and ρ_s_ and ρ_c_ are the shell density material and the core density material, respectively. Thus, the calculated Δ values are 0.20 nm, 0.23 nm, 0.82 nm, and 0.77 nm for MCF-Cit, MCF-Gly, MCF-AuCit, and MCF-AuGly, respectively. Then, the core–shell radius (σ_cs_) can be estimated by applying

(3) $$\sigma_{cs}=\sigma_{c}+2∆$$

Yielding the following sizes, 7.50 nm, 7.55 nm, 8.74 nm, and 8.64 nm, for MCF-Cit, MCF-AuCit, MCF-Gly, and MCF-AuGly, respectively. These results indicate that the Gly-functionalized materials exhibit a slight increase in the size of the organic shell, supporting the magnetic properties of the NMs (as observed in VSM analyses). Due to the thickness of the Gly coating, the Ms decreased from 60 emu/g (CoFe_2_O_4_) to 55 emu/g (MCF-AuGly) because the coating shell interfered with the superficial magnetic interactions, reducing the magnetic response.

### 2.3. Calorimetric Tests

**Figure 6:**
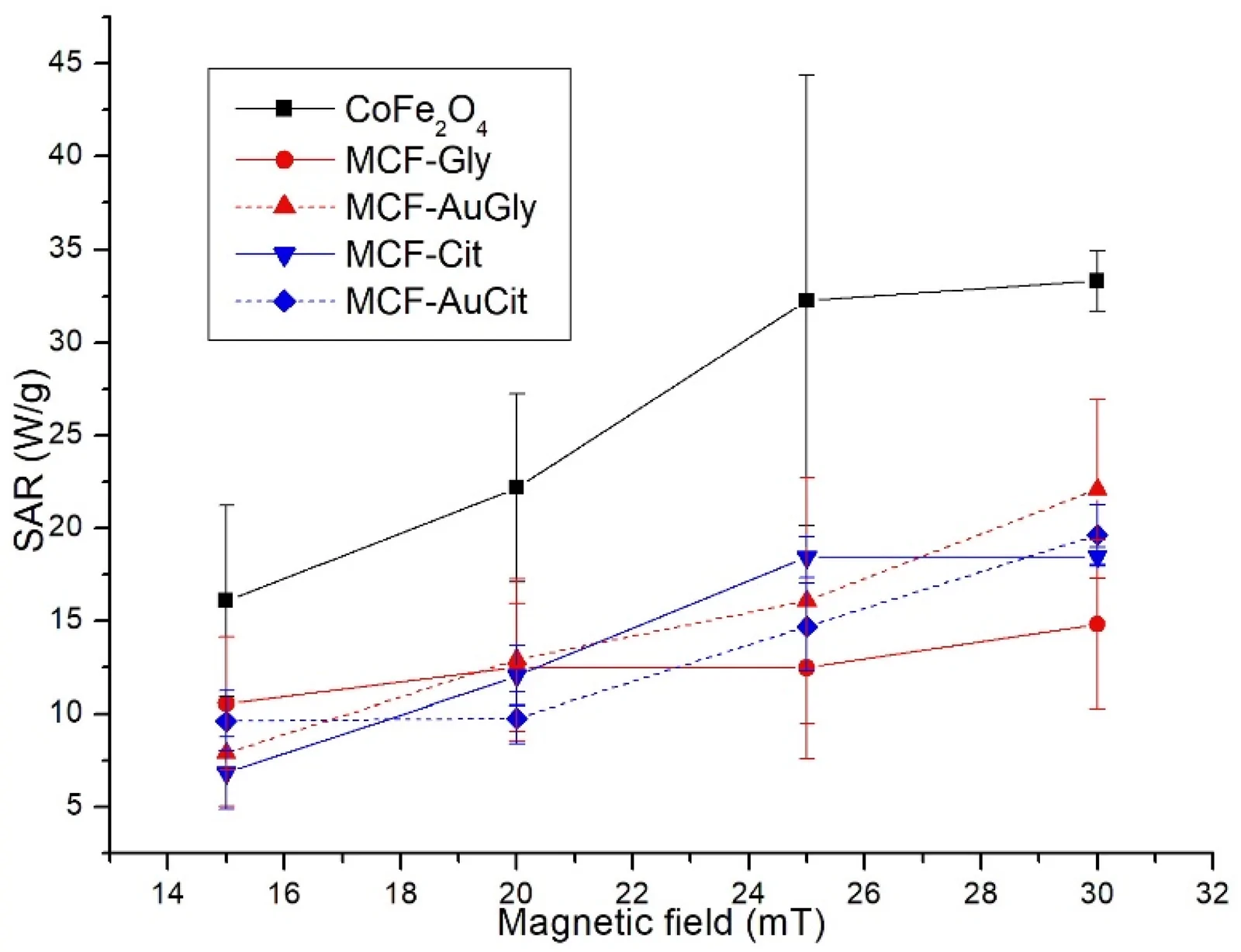
SAR measured as a function of the amplitude of the magnetic field.

Specific Absorption Rate (SAR) analysis was conducted to determine the power density under a variable alternating magnetic field. As presented in Figure 6, pristine CoFe_2_O_4_ demonstrates the highest SAR, a consequence of the absence of an organic shell that would otherwise attenuate its magnetic response. However, the activity of pristine MCF fluctuates considerably due to the limited colloidal stability, which results in nanoparticle aggregation and precipitation. This phenomenon directly diminishes the power absorption capability of a material, which is associated with the increase in the standard deviation [46].

In contrast, functionalized materials (MCF-Gly, MCF-Cit, MCF-AuGly and MCF-AuCit) exhibit a more stable performance during the analysis with a lower deviation in the error bars. As previously discussed, surface-modified NMs present improved colloidal stability that contributes to reducing the dispersion in the error bars (improving the reproducibility). In addition, there are slight differences in the behavior of the NMs due to the nature of the organic coating, with the Cit-modified NMs exhibiting better performance.

In addition, the composites (MCF-Au) under study have a high SAR capability at low magnetic fields. For example, by comparison with Sabale S. et al. (2019) [47], MCF, MCF-AuCit, and MCF-AuGly exhibit enhanced magnetic properties (SAR and Ms) at low concentrations. These results support the advantages of the synthetic approach followed in this study, which renders high-quality nanomaterials for medical applications.

### 2.4. Biocompatibility or Cytotoxic Activity Evaluation

#### 2.4.1. Hemolysis Tests

Human Red Blood Cells (HRBCs) were used to evaluate the biocompatibility of the materials [48]. This test is a fast preliminary assay that renders useful information (the concentration of nanomaterials) that provides the basis for investigating the interaction of NMs with cell lines.

As shown in Table 1 and Figure S9, all the materials are non-hemolytic; a slight increase in the activity of the NMs is observed upon activation (10 min) with IR radiation. It also shows that MCF-AuCit exerts a dose-dependent activity; however, all the NMs under study are non-hemolytic, since the highest percentage of hemolytic activity is 3.66 (MCF-AuGly, 20 μg/mL). These results support the biocompatibility of the NMs with blood cells and their response (ROS formation) to low-energy IR stimulus, followed by disruptive damage to the HRBCs.

The hemolysis assay data indicate that the bioactivity (or cytotoxicity) of MCF-Au NMs occurs at doses higher than 10 μg/mL. This parameter sets the lower limit concentration for the following ROS and viability analyses.

#### 2.4.2. Cell Viability Evaluation

**Figure 7:**
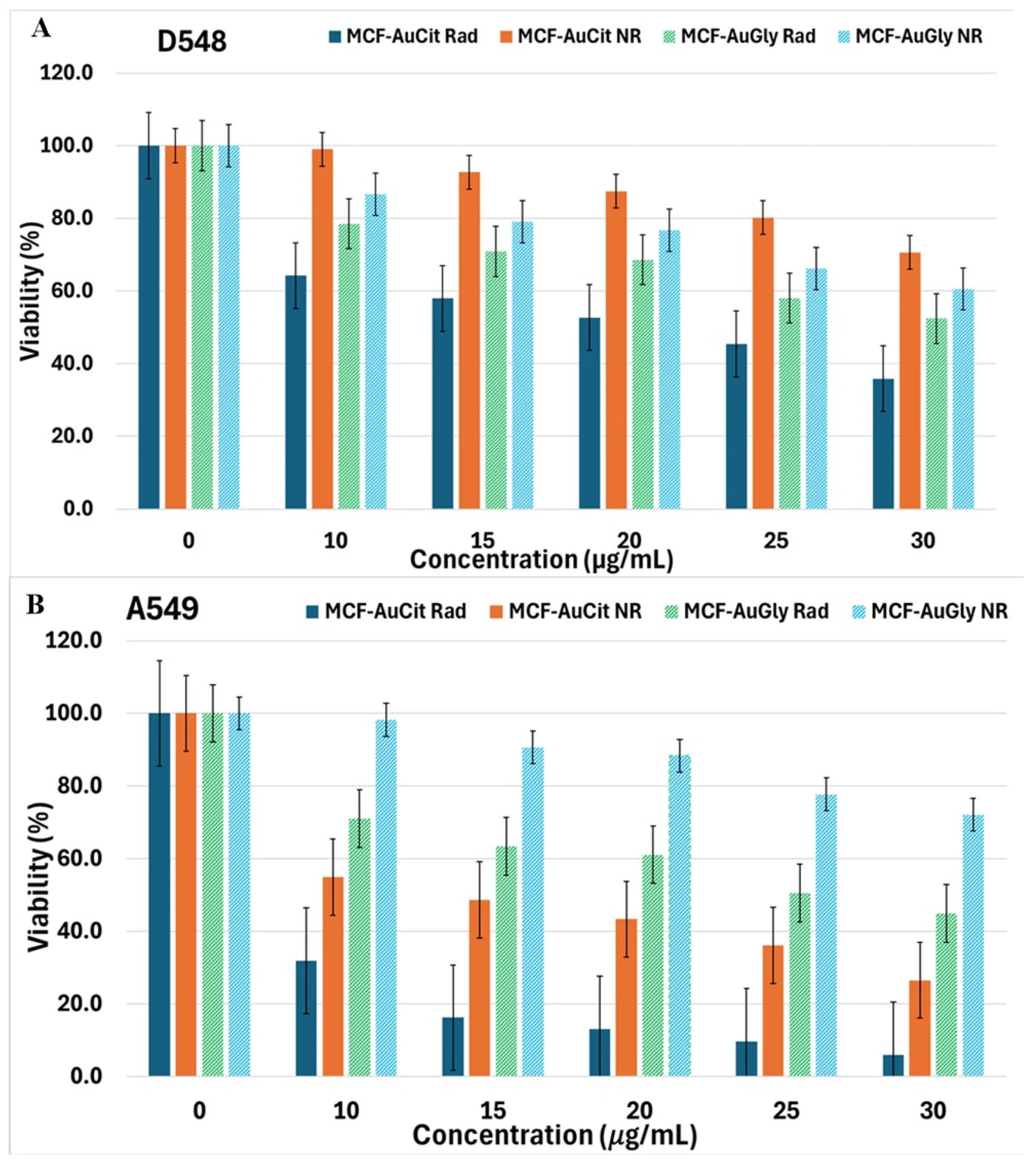
Viability results of the interaction of MNMs with neoplastic (A549) and healthy (D548) cells (radiated and no-radiated assays). (**A**) CCK-8 test with D548 and ferrofluids. (**B**) CCK-8 test with A549 and ferrofluids. There is a remarkable difference in the sensitivity of each cell line to the PDT.

To investigate the biocompatibility or cytotoxic activity of the MCF or MCF-Au NMs, their activity was measured as a concentration-dependent assay (without radiation) and upon activation with IR radiation. Figure 7 presents the viabilities for healthy (D548) and oncogenic (A549) cells. Our results show increased cytotoxic activity in the radiated groups (Table S2). This result confirms the enhanced activity of the MCF-Au NMs upon IR activation (PDT), as expressed by a cytotoxicity enhancement (*p* < 0.05). In the following sections, fluorescence microscopy analysis shows that the cytotoxic activity of the NMs involves oxidative damage (ROS generation).

Figure 7A summarizes the viability of the healthy (D548) cell line after exposure to NMs under different conditions (concentration of NMs, radiated, and non-radiated assays). The NMs exert moderate toxicity against these cells, with higher cytotoxic activity for Gly-functionalized NMs (radiated and non-radiated assays). Furthermore, the groups exposed to Cit-coated NMs maintain higher viabilities (the viabilities keep a value ≥ 80%) at all exposure doses. However, the relative cell viabilities considerably decrease after the activation of the NMs.

Figure 7B illustrates the viabilities for the oncogenic (A549) cell line. MCF-AuCit are the NMs that induce more damage in neoplastic cells at low doses. In addition, the results from Table S2 and Figure 7 indicate that using a dose of 10 μg/mL of these NMs and radiating the cells exposed to the NMs for ten minutes decreases the viability of neoplastic cells (A549) to 31.9%. In contrast, the viability of healthy cells (D548) remains higher (64.2%). In Table S2, the DL50 for the different assays is highlighted in blue; there is always a set of conditions required to reach the DL50 for the A549 cells, whereas D548 cells remain with higher viability percentages. However, it is necessary to maintain a viability greater than or equal to 80% to consider the NMs non-cytotoxic to healthy cells. Under this condition, although MCF-AuCit exerts enhanced cytotoxic activity against A549 cells, the survival of healthy cells is lower than 80%; thus, there is still a need to optimize in vitro exposure conditions to avoid damage to healthy cells.

As previously mentioned, MCF-AuCit exhibits enhanced cytotoxic activity, which can be attributed to the colloidal stability of the NMs, which allows higher contact time with the cells. CCK-8 is based on a mitochondrial probe that helps to quantify living cells; therefore, the viability is measured via mitochondrial pathways. As PDT disrupts these organelles, apoptosis induction is enhanced, resulting in cell death. The CCK-8 results indicate that the viability percentages of the A549 cells are lower than those observed for the D548 cells, with higher cytotoxic activity for the MCF-AuCit NMs. As previously discussed, Cit enhances the colloidal stability of the MCF, which allows higher contact time with the cells and thereby facilitates its uptake [32,49]. As can be seen in the bright field images (Figure S6), both cell lines internalized the nanomaterials after 18 h of exposition and retained them inside, mostly freely, in the cytosol or engulfed them inside a vacuole; internalization of the NMs favored cell destruction by PDT. The cell internalization analysis derived by HTM analysis agrees with fluorescence microscopy assessment, where the A549 cells emit a stronger signal.

Our results demonstrate an efficient platform for PDT, since the NMs under study can be activated using an IR LED lamp (λ = 850 nm) [50,51,52]. Therefore, MCF-Au materials efficiently transfer active species (electrons) to generate ROS, which is responsive to cell damage. These results set the basis for the in-silico optimization of the exposure variables in a more complex biological model (organoid, tumor biopsy, or clinical translation). The recent literature discusses the differences in the activity of NMs when used in vitro or in vivo models. It also advises caution when translating data from in vivo studies using mice to clinical applications in humans [53]. Advanced optical (microscopy) techniques permit us to visualize the entry of NMs into cancerous cells and their cytotoxic mechanisms. The following sections provide additional information on the activity of the NMs using different advanced microscopy techniques.

### 2.5. Evaluation of the Interactions of NMs with A549 and D548 Cells Using Advanced Microscopy Techniques

#### 2.5.1. Atomic Force Microscopy

**Figure 8:**
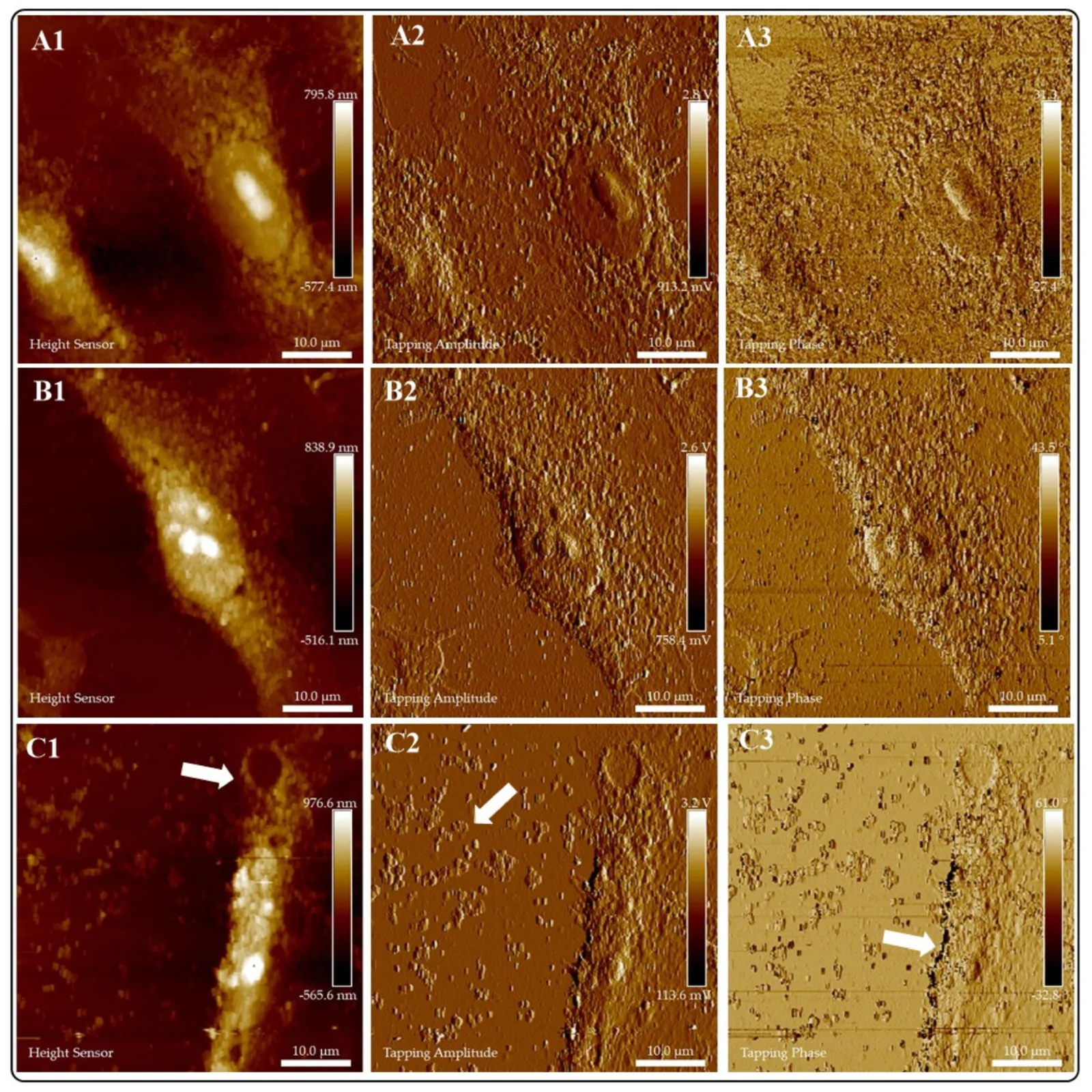
AFM analysis of the interaction of AuNPs and MCF-Au NMs with A549 cells. (**A1**–**A3**) Control group. The cells exhibit characteristic morphology and uniform composition. (**B1**–**B3**) AuNP-treated A549 cells. The cell undergoes morphological changes. (**B3**) The entry of AuNPs into the cells. (**C1**–**C3**) MCF-AuCit-treated A549 cells. The cells exhibit significant morphological changes. (**C1**) The white arrow highlights pore formation. (**C2**) The arrow shows the presence of NMs outside the cells. (**C3**) The arrow points the entry of NMs into the cells. Phase changes demonstrate the presence of NMs inside the cells.

Recent studies remark on the relevance of investigating the interaction of NMs with living cells using modern microscopy techniques. In this work, we use atomic force microscopy (AFM) to observe morphological changes in the cells due to their exposure to MNMs. Figure 8 shows the images of A549 cells. Figure 8(A1–A3) depict the control group (cells with no exposure to NMs). The cells exhibit a regular size and morphology. Figure 8(A3) supports the presence of cells without morphological or compositional alterations. Figure 8(B1–B3) portray the A549 cells exposed to AuNPs. There are differences in the nucleus (an increase in nucleolus) due to Au exposure. In addition, the cells show a slight increase in their width due to Au exposure. Figure 8(B3) supports the entry of Au into the different cell compartments (phase changes).

Earlier studies report the high cytotoxic activity of citrate-stabilized AuNPs (Cit-AuNPs) against MCF-7 cells. Cit-AuNPs exert enhanced cytotoxic activity when compared to starch and Arabic gum-functionalized AuNPs, probably due to the acidic nature of citrate [54]. A different study indicates that Cit-AuNPs can cross the blood–brain barrier facilitated by the bio-interactions of the citrate coat. Furthermore, preceding studies demonstrated that AuNP exposure induces morphological (ballooning) and biochemical (DNA alteration, lipid biogenesis) changes that evolve into cell death (apoptosis or necrosis) [55]. Live-cell imaging allows us to monitor the cell response to the bioactivity of exogenous agents. For example, A549 cells engulf AuNPs in lipid drops to transport them outside the cell [49].

**Figure 9:**
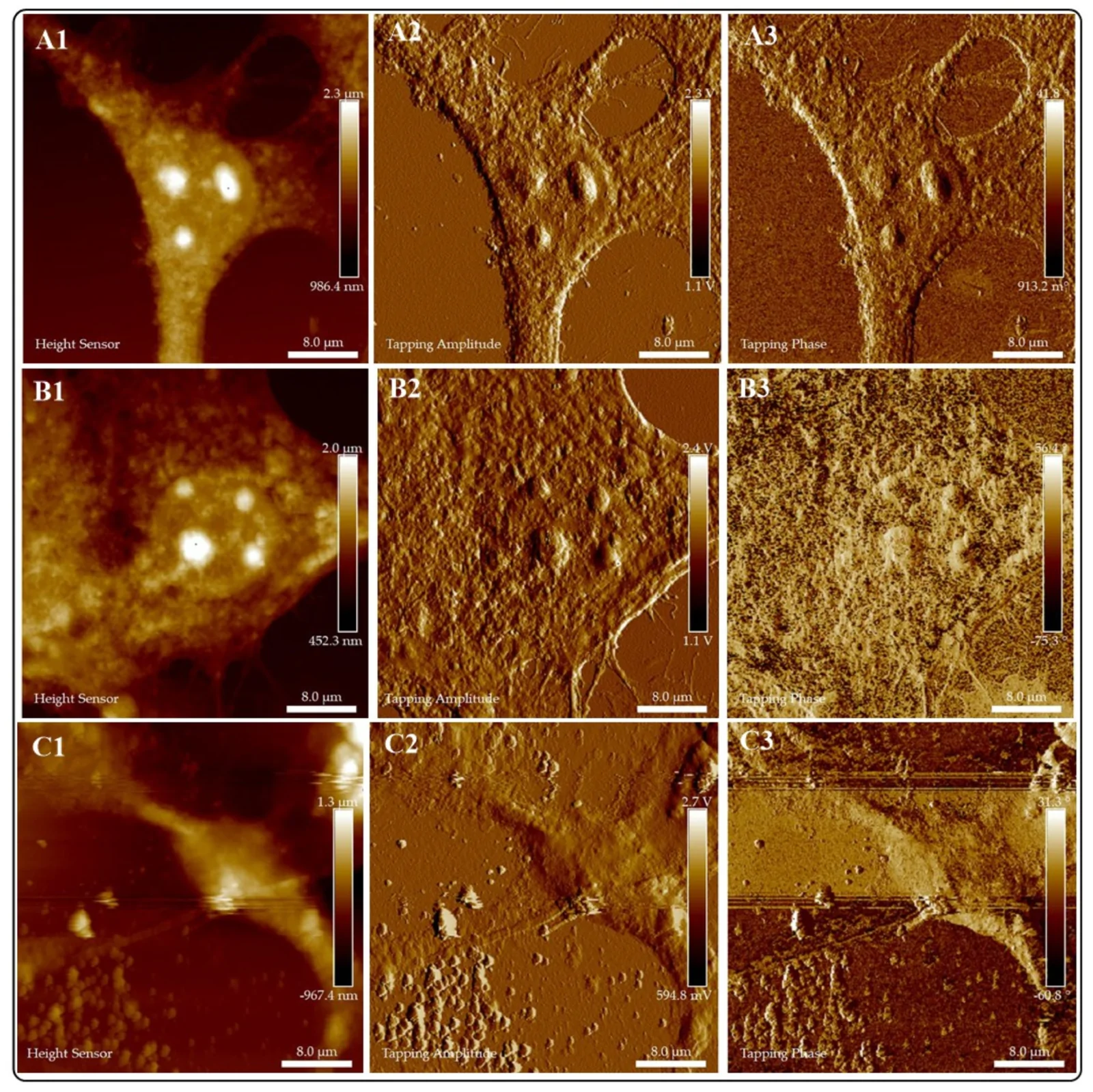
AFM analysis of the exposure of D548 cells to AuNPs or MCF-AuNMs. (**A1**–**A3**) Control group showing typical morphology of D548 cell line. (**B1**–**B3**) Cells exposed to AuNPs, displaying morphological changes and demonstrating the internalization of AuNPs (**B3**). (**C1**–**C3**) Cells exposed to MCF-AuNMs. The image indicates the low penetrability of the NMs into the cells. The NMs cover the cell surface, impeding organelle cell visualization.

Significant morphological changes occur due to exposure to MCF-AuCit NMs. The nuclear region loses definition and the formation of vacuoles or fagosomes occurs in the cytoplasm, disrupting the cell membrane (because of the nature of the AFM technique, it is not clear if the pore is a result of the interaction or mechanical damage due to the presence of NMs on the surface of the cell). Figure 8(C3) illustrates the affinity of the MCF-AuCit to the cell membrane, which facilitates the entry of the NMs into the cell, followed by their enhanced cytotoxicity. Recent studies have discussed the need for the entry of NMs into target cells to improve their therapeutic activity [53]. AFM images (Figure 8(C1–C3) and Figure 9(C1–C3)) show that some MCF-Au NMs internalize into the cells, whereas the others remain outside them (the presence of NMs outside of the cells limits the AFM imaging; these NMs are also responsive to errors in the colorimetric assays of cell viability and can be responsible for deleterious effects during clinical applications).

Figure 9 portrays the images of healthy (D548) cells exposed to AuNPs or MCF-AuCit NMs. The upper line shows the control group (A1–A3). The cells display the typical morphologies of this cell line. There are morphological changes due to AuNP exposure. The biochemical and morphological changes are like those described for A549 cells. In the case of cells exposed to MCF-Au NMs, the presence of NMs on the cell surface interferes with the acquisition of cell images. In addition, the images show the cells covered by NMs, impeding visualization of the cell’s organelles (Figure 9(C1–C3)). Earlier studies report that AuNPs are internalized into cells mainly via clathrin-mediated endocytosis and exit via exocytosis [56]. The improved cytotoxic activity of the NMs under study may be due to increased interactions between the A549 receptors and the NMs, facilitating their internalization. Regarding the entry of MCF-Au NMs, previous studies have reported on the entry and biocompatibility of MnZnFe_2_O_4_-SiO_2_-Au NMs in A549 and A2780 cell lines. However, the biocompatibility assay did not involve activation with IR radiation.

#### 2.5.2. Holotomographic Microscopy Analysis

**Figure 10:**
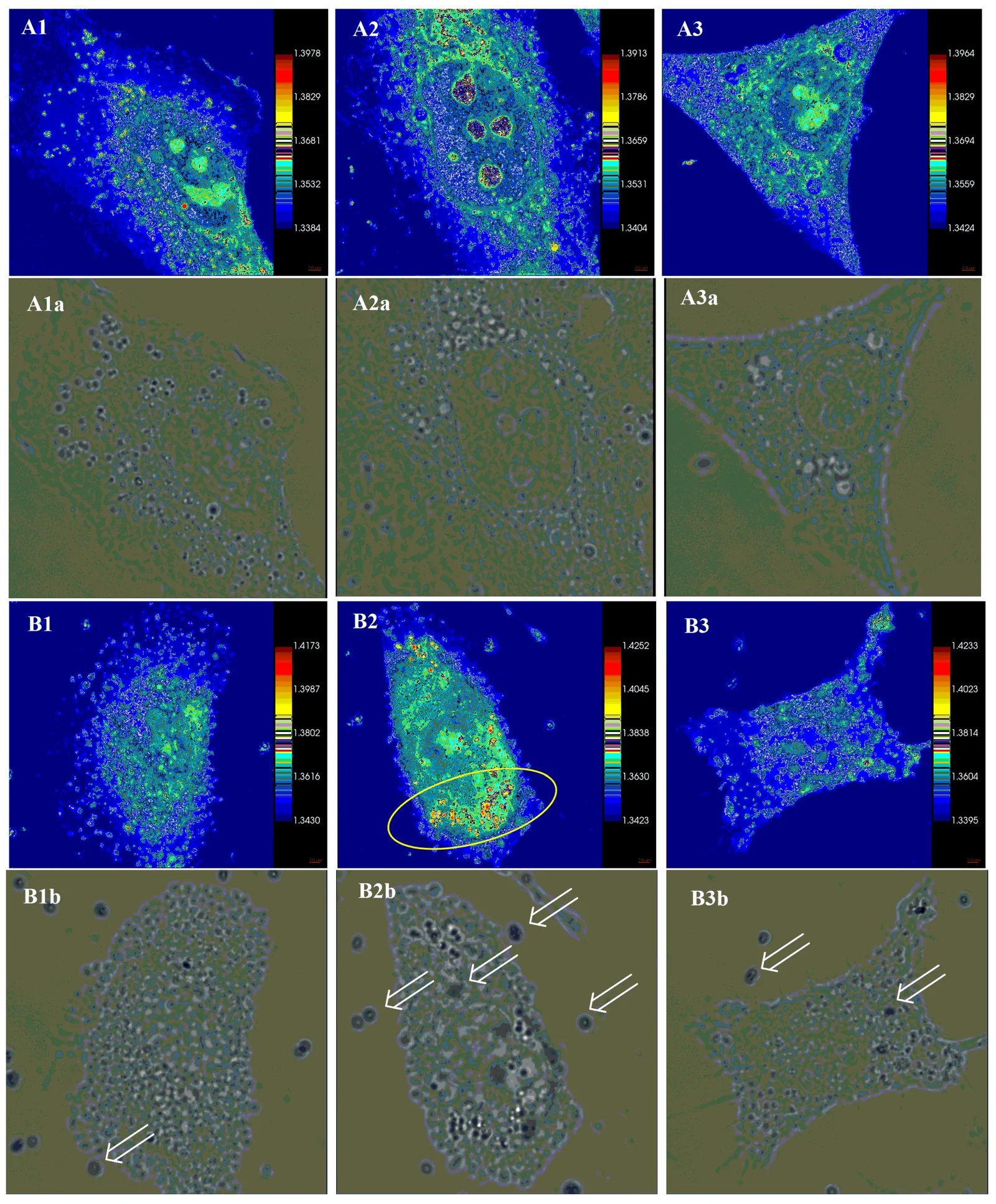
HTM analysis of A549 cells. (**A1**–**A3**) Two-dimensional images (based on the differences between the organelles RI) of A549 cells (control group). (**A1a**–**A3a**) Bright field images of A549 cells (control group). (**B1**–**B3**) Two-dimensional images of A549 cells treated with MCF-AuCit and radiated for 5 min (0.28 A, 850 nm). (**B1a**–**B3a**) BF images of treated A549 cells.

**Figure 11:**
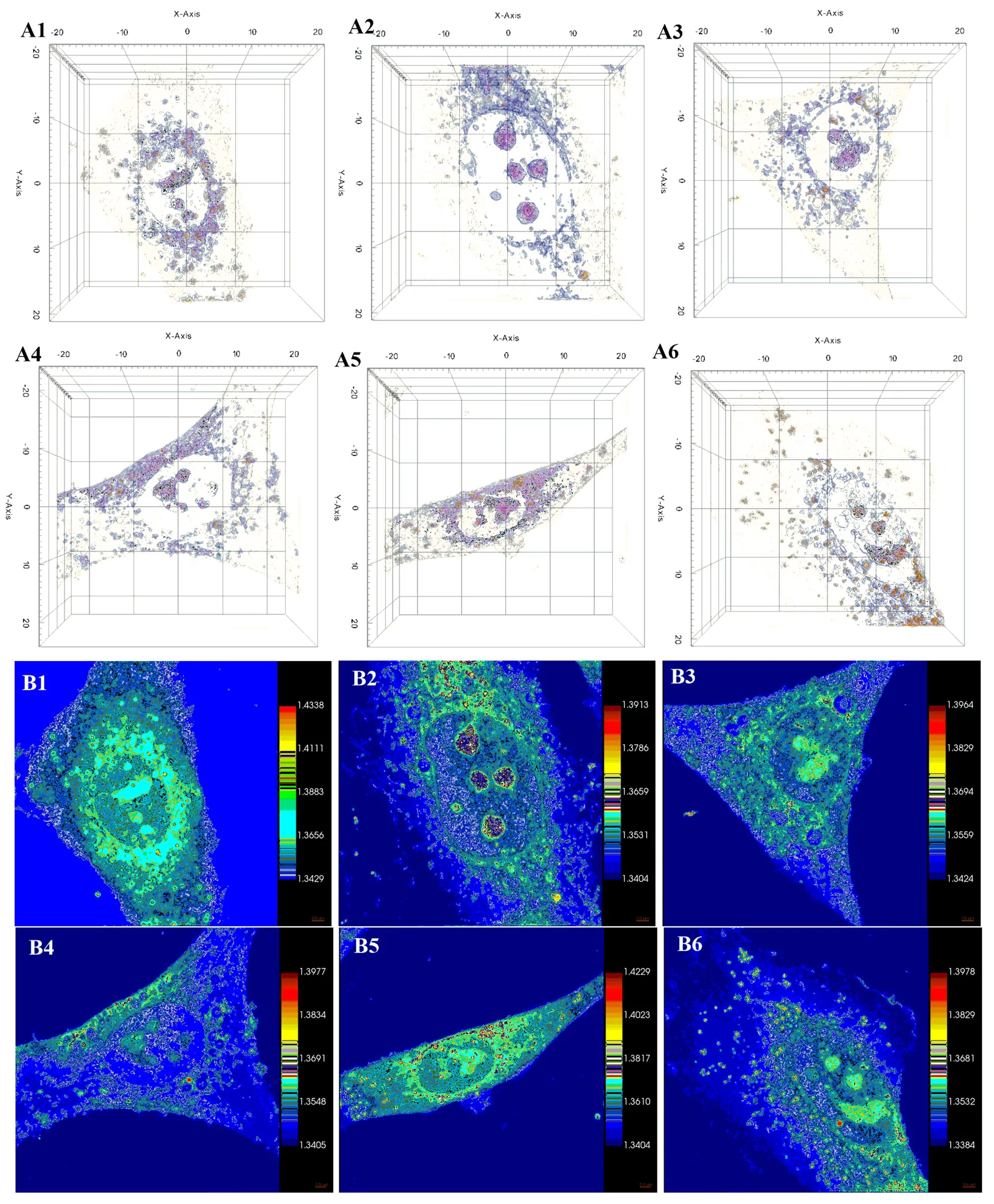
HTM analysis of A549 cells (control group). (**A1**–**A6**) Three-dimensional representation of A549 cells; different colors represent different cell organelles. The cells exhibit typical sizes and morphologies of the A549 cell line. (**B1**–**B6**) Two-diensional representation of A549 cells. Images are based on the differences in the refractive index (RI) of the cell organelles. Bright spots correspond to organelles with a high RI value.

**Figure 12:**
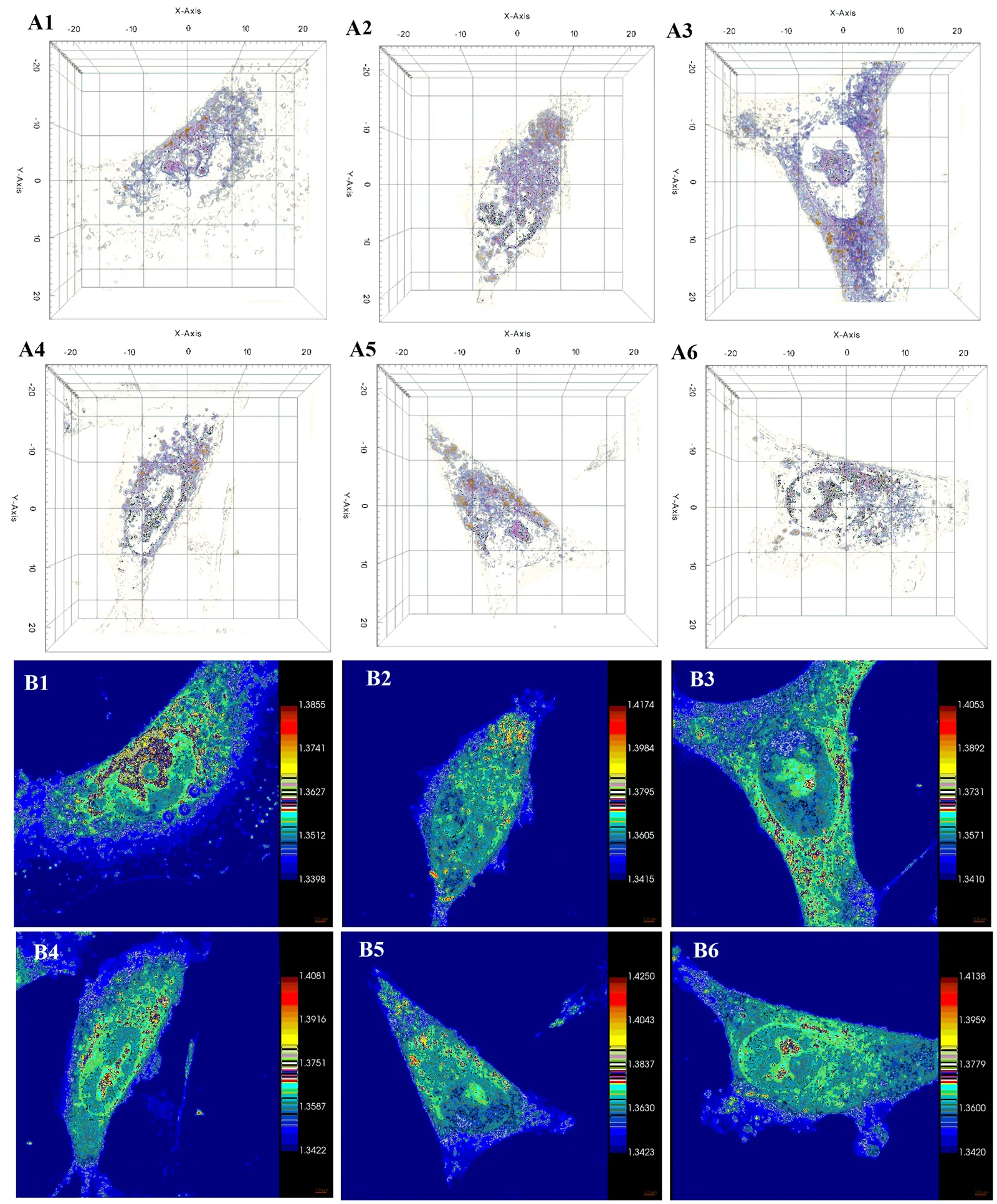
HTM analysis of A549 cells (control group *Radiated*). (**A1**–**A6**) Three-dimensional representation of A549 cells; different colors represent different cell organelles. The cells exhibit typical sizes and morphologies of the A549 cell line. (**A2**) exhibits two cells (division). (**B1**–**B6**) Two-dimensional representation of A549 cells. Images are based on the differences in the refractive index (RI) of the cell organelles. Bright spots correspond to organelles with a high RI value. The cell in (**B1**) shows vacuolization.

**Figure 13:**
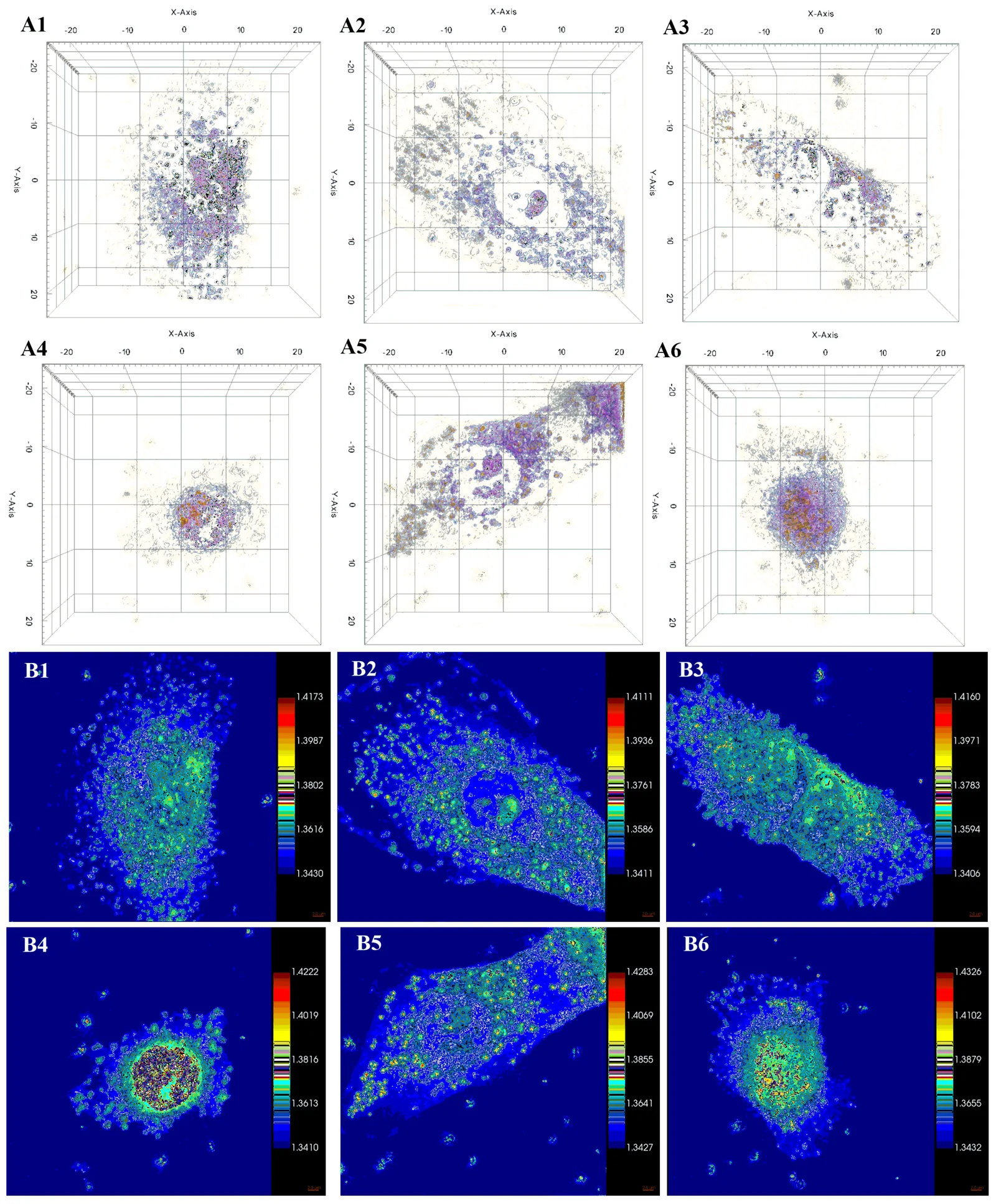
HTM analysis of A549 cells (cells exposed to MCF-AuCit, 1 μg/mL and radiated). (**A1**–**A6**) Three-dimensional representation of treated A549 cells. There are numerous examples of apoptotic cells (**A1**,**A3**,**A4**,**A6**). (**B1**–**B6**) Two-dimensional representation of treated A549 cells.

To complement the AFM analysis, the interaction of NMs with A549 cells was monitored using holotomographic microscopy (HTM) to investigate the entry of the NMs into the cells and the biological response triggered by activation with IR radiation. Our results are illustrated in Figure 10, Figure 11, Figure 12 and Figure 13. Figure 10(A1–A3) depicts the 2D images (based on the differences in the refractive index of the organelles) of A549 cells (control group). The scalar bar shows the different refractive index of the cell components. The cells exhibit different morphologies and sizes. The cells are well-delimited (no damage to nuclear or cellular membranes) and free of exogenous materials. Figure 10(A1a–A3a) portrays the bright-field images of the cells. Additional 2D and 3D images of A549 cells (control group) are presented in Figure 11.

As illustrated in Figure 10(B1–B3), the cells show morphological and biochemical changes upon exposure to MCF-AuCit and IR activation. Figure 10(B1,B1a) depict an apoptotic cell and the presence of NMs in the surrounding media (white arrows). Figure 10(B1,B1a,B3,B3b) show cells, possibly in a necrotic state. Microscopic analysis revealed the morphological features of cell death by necrosis, including multiple cytoplasmic blebs, translucent cytoplasm, cell swelling culminating in plasma membrane rupture, and nuclear dilation. Figure 10(B2,B2a) show an increase in the lipids (bright dots) and a change in their cellular distribution (yellow oval indicating an increase of lipids and a change in their distribution). Late studies support the role of lipid droplets in alleviating cellular stresses; the increased biogenesis of lipid droplets in the cells treated with NMs and IR radiation is a biological response to avoid the oxidative damage of the nano therapy under study [57,58].

Figure 12 shows the 3D and 2D images of the radiated control group. There are no significant differences with the pictures obtained for the no-radiated control group (Figure 11). Some cells show vacuolization (Figure 12(A1,B1)). There is a lack of NMs in the surrounding media; apoptotic features are not predominant, as observed for the cells treated with NMs. In this study, we use low exposure times to IR radiation to avoid the external heating of cells. Preliminary studies investigated the effect of mid-infrared radiation (MIR) in A549 cells. MIR causes the inhibition of cell growth and induces morphological changes in the cells (alteration of the cellular distribution of the cytoskeletal components); in addition, MIR is not harmful to human fetal lung fibroblast cells (MRC5), suggesting a potential role in the use of MIR for lung cancer therapy [59].

Figure 10 and Figure 13 show the HTM analysis of treated cells. Bright field pictures clearly represent the presence of MCF-Au NMs in the media and inside the cells. In addition, necrotic features can be observed in numerous cells. The 3D representation portrays cells with characteristics of necrosis. The massive formation of small surface vesicles illustrated in Figure 13(A1,A3,B1,B3) indicates a loss of membrane integrity with intense cytoplasmic granulation, possibly related to cellular stress; the nuclei show a normal aspect. Figure 13(A4,A6,B4,B6) show rounded morphology, without prolongations and with a reduced cell volume. Features compatible with necrosis (ballooning and membrane rupture); the presence of nuclear condensation; and cell fragments are observed in the surrounding medium. Other researchers have observed similar morphological changes after photodynamic damage to HeLa cells [60].

A general mechanism for the cell damage caused by photodynamic therapy is not well established; however, three main mechanisms of photodamage-induced cell death have been described: apoptosis, necrosis, and autophagy. Regarding cell death by necrosis, some studies indicate that this pathway depends on the intensity of the stimuli (radiation). However, recent investigations demonstrate that necrosis can occur depending on the cellular location of the photosensitizer [61]. Based on the morphological patterns identified with HTM [62], our results indicate a cell death mechanism, possibly due to necrosis; however, future studies will aim to optimize the experimental variables of the therapy for clinical application. Four-dimensional imaging has proven to be an appropriate strategy for modulating the different treatment variables.

#### 2.5.3. Evaluation of the ROS Oxidative Damage Using Fluorescence Microscopy

**Figure 14:**
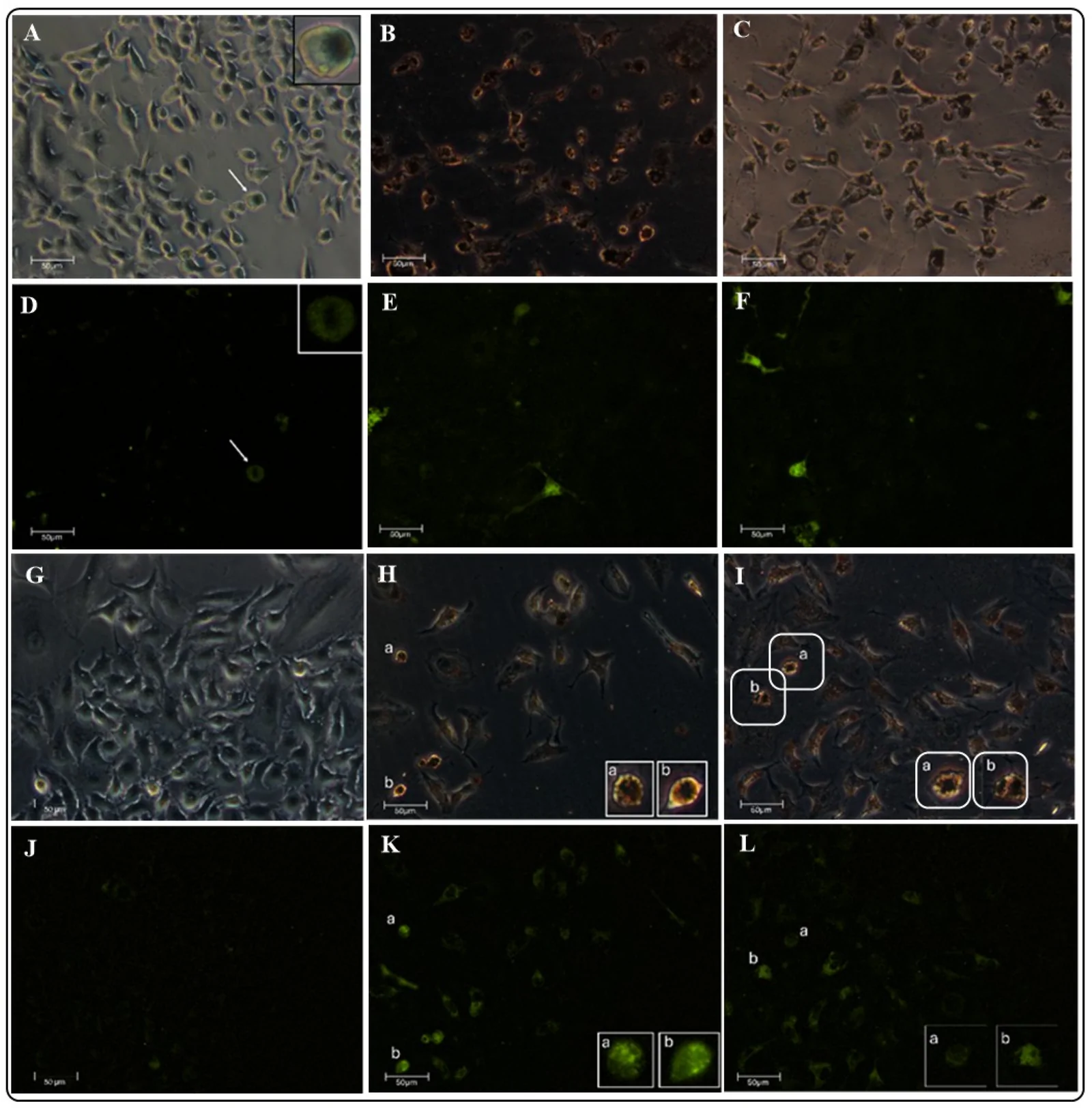
Fluorescence microscopy images of D548 and A549 cells interacting with MCF-AuNMs. (**A**,**D**,**G**,**J**) Bright field (BF) and fluorescence (FL) images of A549 (**A**,**D**) and D548 (**G**,**J**) control cells radiated for 10 min with IR. (**B**,**E**,**H**,**K**) Bright field and fluorescence images of A549 (**B**,**E**) and D548 (**H**,**K**) treated (MCF-AuGly) cells radiated for 10 min with IR. (**C**,**F**,**I**,**L**) Cells treated with MCF-AuCit. As the exposition time increases, fluorescence increases due to ROS production. BF: Bright Field micrography, FL: Fluorescence micrography.

Fluorescence microscopy is a reliable strategy used to investigate the oxidative damage of NMs since the fluorogenic dye, mitoSOX green (MSG), has a higher affinity for superoxide anions (O_2_^−^) than other ROS; therefore, the fluorescence emitted in the analysis can be attributed to ROS formation. The O_2_^−^ is formed by NADPH oxidases (NOX4) in the cell nucleus and inside the mitochondria as a product of the electron transport chain [63]. Excessive ROS production can lead to membrane disruption and Cytochrome C liberation, which leads to Caspase 9 formation and apoptosis induction [64]. In this study, ROS production may be attributed to the activity of an exogenous agent (NMS), which results in cellular stress induction. After 18 h of exposition, both lines internalize the nanomaterials, showing no membrane disruption or morphological changes (Figure 14B,C,H,I) compared with the control groups (Figure 14A,G). In addition, fluorescence microscopy data indicate that there is no cellular damage resulting from exposure to IR radiation (10 min). The D548 and A549 cells (control groups) preserve their normal morphology (Figure 14 insert A and D, showing a mitotic cell) and exhibit a mild fluorescent signal (Figure 14D,J) due to the normal cell metabolism [65].

Furthermore, fluorescence microscopy indicates differences in the bioactivity of MCF-Au and MCF (Figures S10–S14) NMs. Figure S10 demonstrates that AuNP enhances the fluorescent signals due to the ROS production via LSPR (Figure S12(B2,C2)). MCF-Au NMs respond to IR (850 nm) activation and exert cytotoxicity, supporting their feasibility as PDT agents. Nevertheless, there is a significant difference in the fluorescence signal distributions of the different NMs under study after activation with IR radiation. For example, MCF-Cit (Figure S10(C1)) exhibits strong focused fluorescence (Figure S10(C2)), whereas MCF-Gly (Figure S10(B2)) has a discrete fluorescence (points) distributed throughout the sample.

Compared with the control groups (Figure 14D,J, Figures S11(C2), S12(C2), S13(C2) and S14(C2)), after 10 min of radiation, the fluorescent signal increases in both cell lines (Figure 14E,F,K,L), demonstrating the ROS enhancement that can be correlated to the viability decrement in the viability assays [60,66]. In addition, the cell’s morphology indicates intrinsic apoptosis (mitochondrial pathway), induced by PDT-mediated oxidative damage [51,67,68,69,70,71]. There is a standard criterion by which in vitro cells can be classified as apoptotic or necrotic cells [60] based on their morphological characteristics and the ROS presence, related to mitochondrial pathways [51,68,72,73,74]. As in Figure 14C,I, there are groups of cells that preserve their morphology, showing no membrane disruption or damage; however, there are visible groups of rounded cells [40] with a stronger fluorescent signal (Figure 14F,L), chromatin condensation (Figure 14H,K, inserts a and b), and cell shrinkage, characteristics of apoptosis [60,64,71,75,76,77,78]. Therefore, cell death is enhanced by a caspase-mediated route rather than necroptosis or physical damage caused by MCF-Au.

Apoptosis is a desirable cell death pathway due to its localized effect, which avoids secondary effects, as well as its immunogenic synergistic effects. As cells liberate the apoptotic residuals as CX3CL1, phagocytic cells are attracted, leading to an immunogenic detection of the tumoral masses and reduction enhancement [79]. Therefore, PDT can potentially enhance classical therapies by inducing cell death and promoting immunogenic chemotaxis, thereby acting as an adjuvant treatment.

## 3. Materials and Methods

### 3.1. Synthesis of CoFe_2_O_4_

The synthesis of magnetic nanoparticles was produced using a microwave-assisted solvothermal route following a previously published protocol [1]. In a microwave reactor (CEM Discover System 908005), 0.33 mmol of Cobalt (II) acetylacetonate (Aldrich, Saint Louis, MO, USA. Product of France) and 0.66 mmol of Iron (III) acetylacetonate (Aldrich, Saint Louis, MO, USA) were dissolved in 5 mL of benzyl alcohol (Karal, León, Gto., Mexico). The reaction consisted of two cycles; the first took 2 min at 60 °C and 40 W. The second step took 5 min at 200 °C and 300 W of power. Later, the material was washed with ether while it was decanted using a permanent magnet and then it was dried at 60 °C.

#### 3.1.1. CoFe_2_O_4_ Functionalization

Surface functionalization was performed using sodium citrate dihydrate (J.T. Baker, Radnor, PA, USA. Product of Canada) or glycine (J.T. Baker, Radnor, PA, USA). In a round-bottom flask, we added 2.15 × 10**^−^**^4^ moles of citrate and dissolved them in 80 mL of deionized water (DI) (ethanol for glycine). Then, 4.30 × 10**^−^**^4^ moles of CF were added and sonicated for 10 min in the solution. Afterwards, the suspension was heated at 60 °C for 2 h with stirring. Finally, the CF-functionalized nanoparticles were separated and centrifuged at 4500 rpm for 10 min.

#### 3.1.2. Au Nanoparticle Synthesis

The synthesis of AuNPs proceeded according to a previously published (2019) protocol with slight modifications [28]. In brief, in 20 mL of deionized water (DI), 40 µL of a 50 mM HAuCl_4_·3H_2_O (Sigma Aldrich, Saint Louis, MO, USA) solution was added and mixed with 0.5 mL of sodium citrate 0.1% *w*/*v* and 100 µL of H_2_O_2_ 30% (215 mM) (REASOL, Iztapalapa, CDMX, Mexico) with vigorous stirring at 20 °C. The solution turned slightly yellowish, then it became colorless, and after approximately 7 min, it changed to pink. Finally, the colloid was stirred for 30 min to ensure a complete reaction.

#### 3.1.3. AuNP Deposition on MCF-Cit and MCF-Gly (MCF-AuCit and MCF-AuGly)

Using the AuNP suspension created above, 10 mg of MCF-Cit was suspended in 1 mL of AuNP colloid. The mixture was then sonicated for 5 min and stirred for 24 h at room temperature. Then, the suspension was centrifuged for 10 min at 5000 rpm and washed twice with DI. MCF-Au composites were decanted using a magnet and dried in the oven at 60 °C.

### 3.2. Characterization

XRD analysis was performed using a Siemens D500 diffractometer (Siemens, Karisruhe, Germany) with a Cu-Kα source (λ = 1.541874 Å), a 2θ angle variation between 20° and 80°, and s step size of 0.02°. A Jeol Arm 200F (Tokyo, Japan) microscope was used for TEM analysis. The instrument was operated at 200 kV. We used a cold field emission gun (Cold FEG), the Schottky-type and spherical aberration (Cs) corrector CESCOR in STEM mode with a resolution of 80 pm, and high-angle annular dark-field (HAADF) imaging (Z contrast), together with an EDS Oxford AZtecTEM detector for high-resolution chemical analysis. VSM was carried out on a Versa-Lab Vibrating Sample Magnetometer (Quantum Design, San Diego, CA, USA). FTIR analysis was performed on a JASCO FT/IR-4100, and UV-Vis absorption spectra were obtained in a Thermo Scientific Helios Omega UV-Vis spectrophotometer (Thermo Scientific, Walthman, MA, USA) TGA was conducted using a TGA-1000 (Instrument Specialists Inc., Twin Lakes, WI, USA) that swept from 0 to 1000 °C at 10 °C/min.

### 3.3. Calorimetric Analyses

To measure the SAR of the MCF-Aus, a previously reported magnetic–calorimetric system [80] (MX patent 65340) was employed. The equipment had a frequency range of 185 kHz < f < 530 kHz and a magnetic field range of 0 < H < 366 Oe. The power density was quantified using alternating magnetic fields at a 530 kHz frequency for 5 min from 15 to 30 Oe. The sample’s temperature was measured using a fluoroptic sensor, Luxtron-One (Luxtron Corporation, Santa Clara, CA, USA). Samples were measured by creating a ferrofluid using Arabic gum 3% and using a 1 mg/1 mL concentration of each MCF-Au.

### 3.4. Biocompatibility or Cytotoxic Activity Evaluation

#### 3.4.1. Hemolysis Assay

The hemolytic activity of the NMs was examined as a preliminary biocompatibility test via a previously published protocol [58]. In particular, it was performed following the ASTM F756-13 standard (Standard practice for assessment of hemolytic properties of the materials). ASTM International (2017). ASTM F756-17: Standard practice for assesssment of hemolytic properties of Materials. ASTM International. The blood samples were obtained from three healthy donors (with no diagnosed chronic diseases, aged 20–30 years, and with a minimum fasting period of 6 h). Heparin-stabilized human blood was freshly collected. Afterwards, 10 mL of physiological saline solution (PSS) was added to the test tubes. Then, a corresponding quantity of nanomaterials in the colloidal form was suspended in Arabic gum solution (3% *w*/*v*). Finally, 100 µL of whole blood was added to each tube. The tubes were incubated at 37 °C in a water bath at 65 RPM for three hours.

The same methodology was used to evaluate the effect of light activation (Photodynamic therapy). The samples were exposed for 10 min to IR light (850 nm) to evaluate changes in the hemolytic activity of the NMs due to the PDT.

#### 3.4.2. CCK-8 Assay

In addition to the hemolytic activity of the NMs under study, the viability of healthy (Detroit 548 CCL-116) and cancerous (A549) cells after exposure to NMs was investigated using the CCK-8 assay. For this, 10 mL of the CCK-8 reagent was added directly into each well and incubated for 2 h at 37 °C protected from light. The absorbance was measured at 450 nm using a microplate reader (STAT FAX 4200; Awareness Technologies USA, New York, NY, USA).

### 3.5. Evaluation of the Interactions of NMs with A549 and D548 Cells Using Advanced Microscopy Techniques

#### 3.5.1. Atomic Force Microscopy (AFM) Analysis

AFM analysis was conducted to evaluate the effects (morphological changes and the entry of NMs) of exposure to AuNPs or MCF-Au NMs in neoplastic (A549) and healthy cells (D548). The protocols used for cell preparation (control and exposed to NMs) for AFM analysis have been reported by our research group. In brief, the cells were grown in a 24-well plate using DMEM BFS 10% medium and incubated for 48 h at 37 °C under a CO_2_ atmosphere (to a concentration of 100,000 cells/cm^2^ per well). The control group was then separated and fixed in 10% buffered paraformaldehyde and dried with ethanol at increasing concentrations. The treated cells were exposed to AuNPs or MCF-Au NMs (10 μg/mL) for six hours and then treated with IR light (850 nm) for 10 min before incubation for additional 12 h. Finally, the cells were fixed using the same procedure as employed for the control cells. All cells were analyzed using a Scanasyst microscope (Bruker, Santa Barbara, CA, USA. Tapping mode, air) with a probe model OTESPA-R3 (f_o_: 300 kHz). The images were recorded according to the following parameters: (a) scan size—50 or 25 μm; (b) scan rate—30 μm/s; (c) samples/line—512; (d) lines—512.

#### 3.5.2. Holotomographic Microscopy (HTM) Analysis

The A549 cells were cultured in DMEM medium, as previously described, to evaluate the effects (morphological changes and the entry of NMs) of exposure (and activity of NMs upon activation with IR) to AuNPs or MCF-Au NMs in neoplastic (A549) cells in real time (4D imaging). Cells were seeded in HTM plates and allowed to reach adherence for 48 h before NM exposure, whereas the control group was not exposed to NMs but was allowed to incubate for a further 24 h. After that, the cells were exposed (6 h) to AuNPs or MCF-Au NMs (1 μg/mL). Later, all dishes were analyzed using a holotomographic microscope (Tomocube, Yuseong-gu, Daejeon, Republic of Korea) integrated with an IR laser (850 nm) for the real-time monitoring of photodynamic therapy. The cells were irradiated for 5 min and analyzed by HTM. Three-dimensional images of the cells were reconstructed using HTM’s software (TompStudio^TM^. Ver. 3.0). To interpret the 3D RI tomograms, cell components and NMs are segmented using thresholding based on RI values.

#### 3.5.3. Evaluation of the ROS Oxidative Damage Using Fluorescence Microscopy

To investigate the oxidative damage of the materials, ROS quantification proceeded using the fluorogenic dye MitoSOX Green (MSG) (Thermo Fisher, Life Technoligies Corporation, Carlsbad, CA, USA), which is highly selective for reactive oxygen species (ROS). The probe efficiently absorbs and emits its signal at 488 and 510 nm, respectively. It is a high-affinity mitochondrial probe that is oxidized by superoxide (O_2_^−^) and emits green fluorescence. Cells were seeded in a 24-well plate at a density of 97,500 cells/cm^2^ per well. The plate was divided as follows: (a) control group (only cells in the plate), (b) cells exposed to MCF-AuCit, and (c) cells exposed to MCF-AuGly. Each assay was conducted in triplicate. Cells were exposed to 0.1 mg/mL of each MCF-Au. The control cells (DMEM BFS 1%) and treated (DMEM BFS 1% + MCF-Au) cells were incubated for 18 h. Later, the medium was removed, and each well was washed 3 times with PBS. Then, the MitoSOX Green 1 µM in N,N-dimethylformamide was added and incubated for 1 h. After incubation, the cells were irradiated with an IR lamp (850 nm) at different time intervals (1 min, 5 min, and 10 min). The cells were then imaged using a fluorescence microscope.

### 3.6. Statistical Analysis

All experiments were performed in triplicate with three individual runs to obtain statistical data (N = 9). For all the statistical tests, an analysis of variance (ANOVA) model was applied, though to consider trust value statistically significant, α = 0.05 was considered (95% confidence). Statistical analysis was performed using Tukey’s test; this multiple comparison method is used to identify significant differences between groups (*p* < 0.05).

## 4. Conclusions

We can establish that MCF-Au NMs exhibits suitable properties that can be further investigated to create efficient alternatives capable of improving classical antineoplastic treatments. The MNMs act upon different stimuli, responding to alternating magnetic fields (magnetic hyperthermia) and generating ROS via LSPR phenomena (PDT). The synthesis protocol of MNMs generates a highly crystalline, homogenous, and strongly responsive material to the magnetic fields capable of generating HT. In addition, it is a chemically stable material capable whose surface can be modified via a simple functionalizing protocol. Therefore, MCF serves as a base for a wide range of nanostructured platforms due to its ability to incorporate different materials using easy and low-cost methodologies. In addition, AuNP was synthesized using a one-pot green route and creating multibranched structures with an LSPR in the first biological window. The NM’s response to optical stimuli (IR radiation) results in the formation of ROS (as demonstrated using the MTG probe) and reduced cell viability in cancerous cells. The A549 line exhibits a higher level of sensitivity to PDT than D548 due to the enhanced interaction of NMs with cell receptors, which can be correlated with the ROS concentration inside the cell, leading to intrinsic apoptosis. Additionally, the MCF-Au NMs exhibit acceptable biocompatibility with healthy cells (RBCs, D548). Moreover, this is the first study to use a modified HTM microscope to study in real time the interaction of MCF-AuNMs upon activation with IR light (PDT). Therefore, we present this investigation as evidence that the MCF-Au based on MCF and AuNPs is a novel and promising platform with which to apply hybrid therapy (HT and PDT) as an adjuvant to classical anticancer therapies. Further studies aim to optimize the interaction (time of exposure, dose, and time of radiation) between neoplastic cells and MCF-Au NMs, using both in vitro and in silico studies to facilitate their clinical application.

## Figures and Tables

**Table 1:** Results of hemolysis percentage after 3 h (with 10 min of IR light irradiation) of the hemolysis assay using the ferrofluids.

Material	10 µg/mL	15 µg/mL	20 µg/mL	25 µg/mL	30 µg/mL
MCF NR	0	0	0	0	0
MCF R	0	0	0	0	0
MCF-AuCit NR	0	0	0	0	0
MCF-AuCit R	0	0	1.7	2.0	2.0
MCF-AuGly NR	0	0	0	0	0
MCF-AuGly R	0	0	3.7	1.2	1.0

NR: not radiated; R: radiated.

## Data Availability

The original contributions presented in this study are included in the article/Supplementary Material. Further inquiries can be directed to the corresponding authors.

## Supplementary Materials

The following supporting information can be downloaded at: https://www.mdpi.com/article/10.3390/ijms26167732/s1.

## Abbreviations

The following abbreviations are used in this manuscript

L	Ligand (functionalization molecule)
Cit	Sodium citrate
Gly	Glycine
MCF	Magnetic cobalt ferrite (CoFe_2_O_4_)
NMs	Nanomaterials
MCF-Au-L	Multifunctional plasmonic nanostructured materials
Au NPs	Gold nanoparticles
PDT	Photodynamic therapy
LSPR	Localized surface plasmon resonance
PTT	Photothermal therapy
PCE	Photoconversion Energy
A549	Lung alveolar cancer cells
Detroit 548	Skin fibroblasts cells
HR-TEM	High-resolution transmission electron microscopy
EDX	Energy-dispersive X-ray spectroscopy
UV-Vis	Ultraviolet-visible
LSP	Localized surface plasmon
FT-IR	Fourier transformed-infrared spectroscopy
TGA	Thermogravimetric analysis
CCK-8	Cell Counting Kit-8
AFM	Atomic force microscopy
CFM	Confocal fluorescence microscopy
HTM	Holotomographic microscopy
MHT	Magnetic hyperthermia therapy
MNMs	Magnetic nanomaterials
DNA	Desoxyribonucleic acid
ROS	Reactive oxygen species
