# Supplementary material for: Evaluation of CoFe2O4-L-Au (L: Citrate, Glycine) as Superparamagnetic–Plasmonic Nanocomposites for Enhanced Cytotoxic Activity Towards Oncogenic (A549) Cells
Source: Int J Mol Sci. 2025 Aug 10;26(16):7732. doi: 10.3390/ijms26167732 (PMC12386403; doi:10.3390/ijms26167732)
Supplement: Supplementary file 1 [file ijms-26-07732-s001.zip › ijms-3776898-supplementary.pdf]

Supplementary Information

Evaluation of $CoFe_2O_4$-L-Au (L: Citrate, glycine) as superparamagnetic-plasmonic nanocomposites for enhanced cytotoxic activity towards oncogenic (A549) cells.

Alberto Lozano-López[1], M. Cano-González[2], J. Ventura-Juárez[3], M H Muñoz Ortega.[1], Israel Betancourt[4], Juan Antonio Zapien[5*], Iliana E. Medina-Ramirez[1*]

[1]Departamento de Química, Centro de Ciencias Básicas, Universidad Autónoma de Aguascalientes, Av. Universidad # 940, C.P. 20100, Aguascalientes, Aguascalientes, México,
[2]Centro Universitario de la Ciénega Universidad de Guadalajara, Av. Universidad #1115, col. Linda Vista, C.P 47810, Ocotlán, Jalisco, México.
[3]Departamento de Morfología, Centro de Ciencias Básicas, Universidad Autónoma de Aguascalientes, Av. Universidad #940, C.P. 20100, Aguascalientes, Aguascalientes, México.
[4] Instituto de Investigaciones en Materiales, Universidad Nacional Autónoma de México, México.
[5] Department of Materials Science and Engineering, City University of Hong Kong, Hong Kong SAR, PR China.

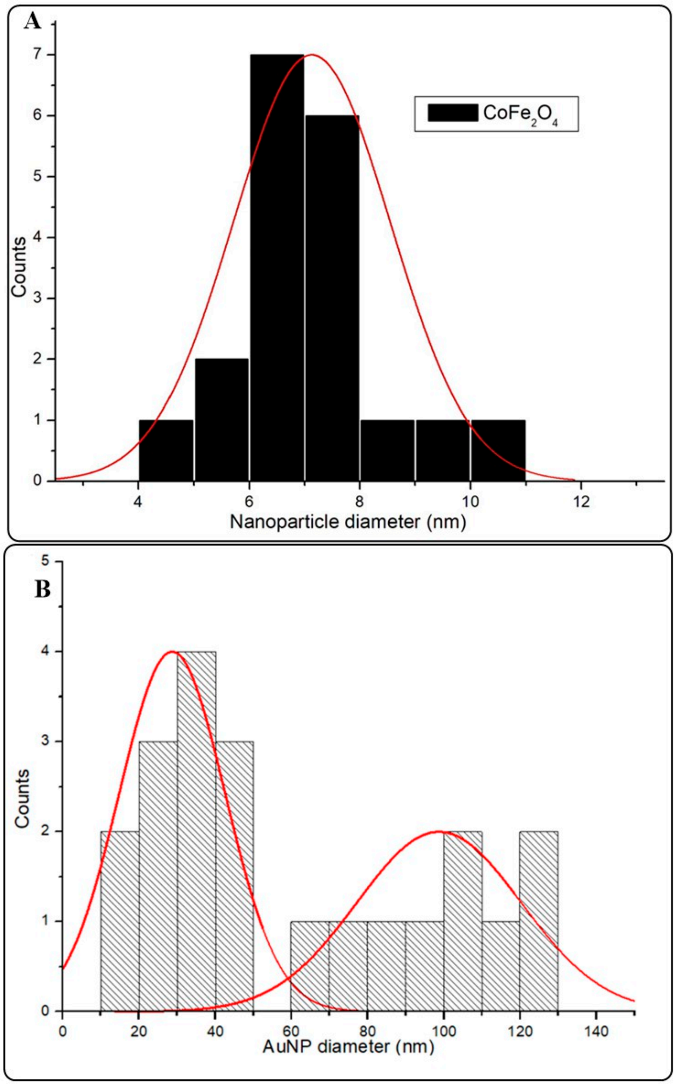

Figure S1. Size distribution (diameter) of $CoFe_2O_4$ (**A**) and Au NPs (**B**).

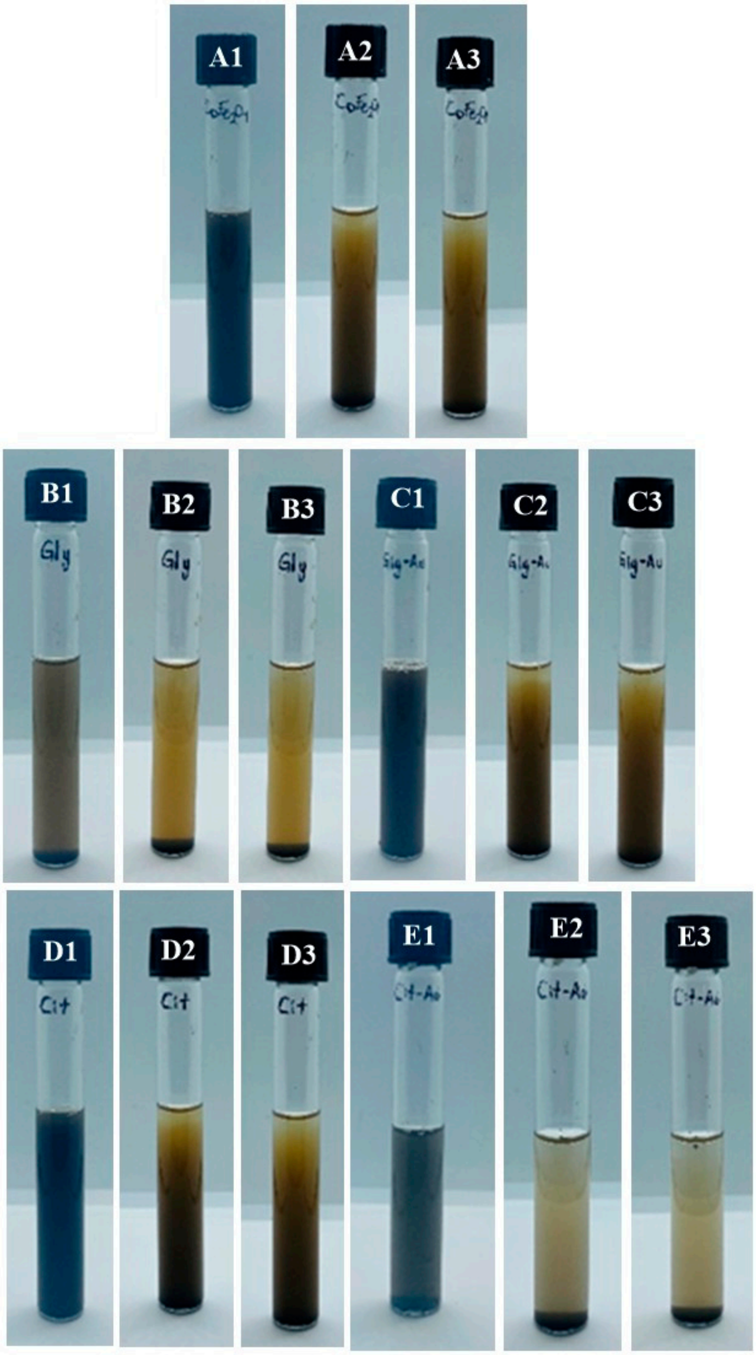

Figure S2. Colloidal stability test of the ferrofluids (Arabic gum 3%) at a concentration of 1mg/mL, and the pictures were acquired at 0,16 and 24 hours (labeled as 1, 2, and 3, respectively). A) $CoFe_2O_4$ (MCF) B) MCF-Gly C) MCF-AuGly D) MCF-Cit E) MCF-AuCit .

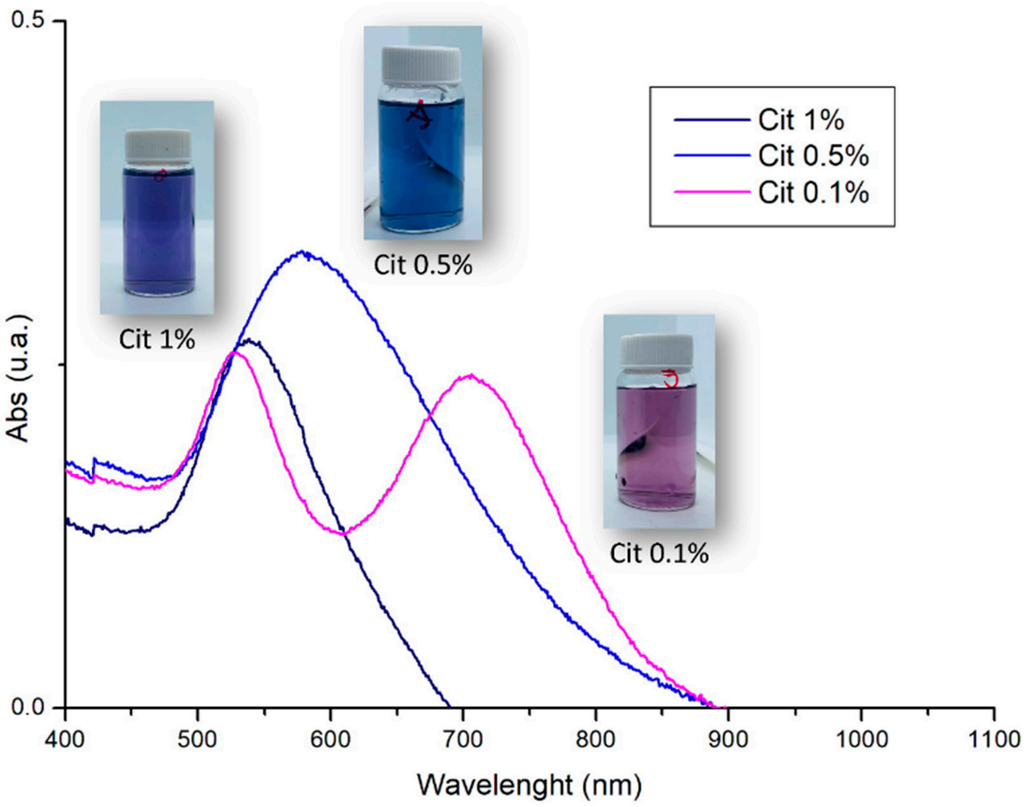

Figure S3. UV-Vis spectra of AuNP's synthesized using different Cit concentrations showing the LSPR modification. The color of each spectrum was matched with the synthesized colloidal gold suspension.

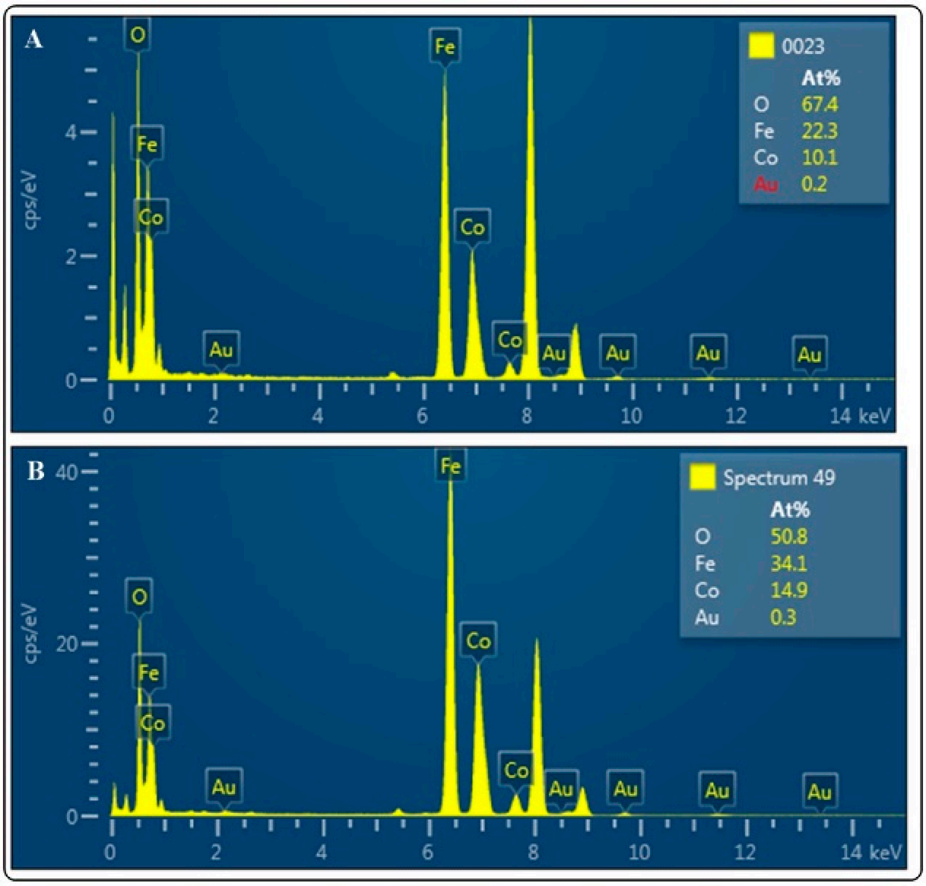

Figure S4. EDX analysis of MCF-Au NMs. **A)** MCF-AuGly, **B)** MCF-AuCit. The EDX analysis confirms the formation of cobalt ferrite-Au composites. There is a low amount of gold on the surface of magnetic NMs.

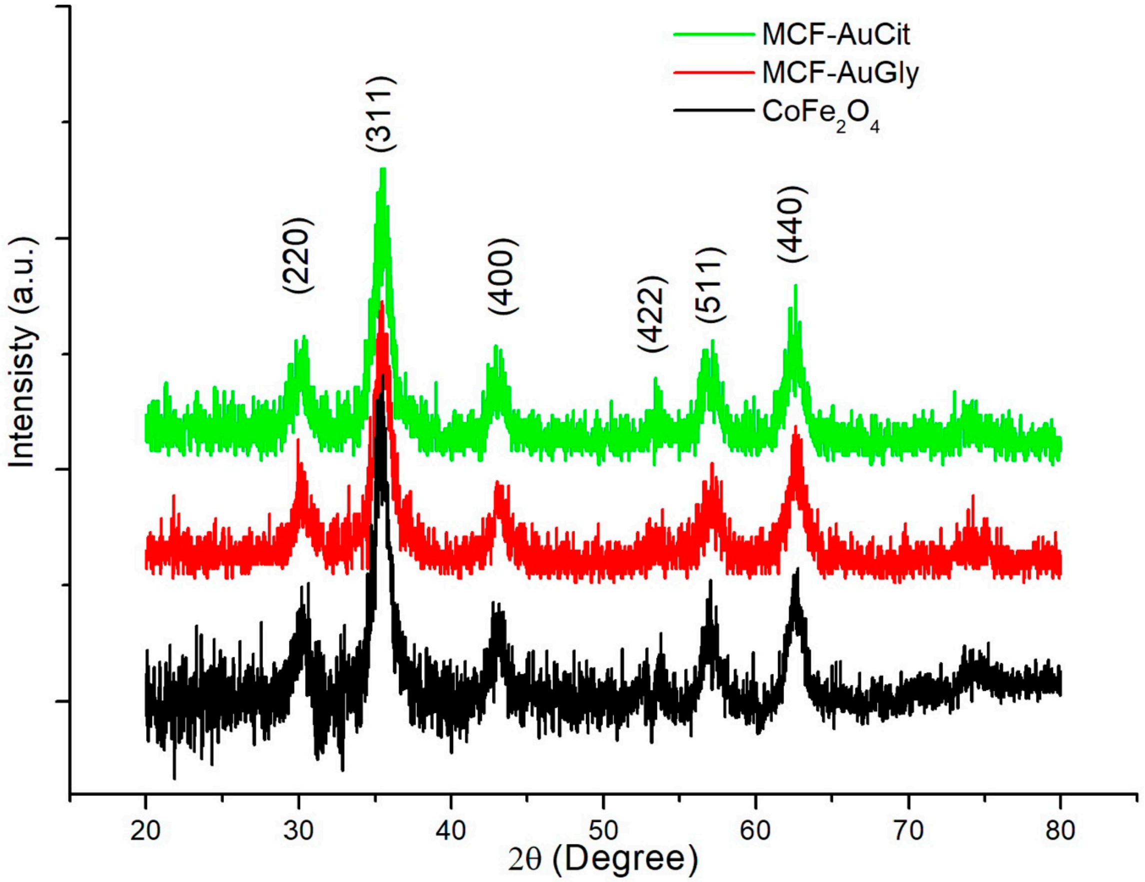

Figure S5. XRD spectra of pristine MCF, MCF-AuCit, and MCF-AuGly.

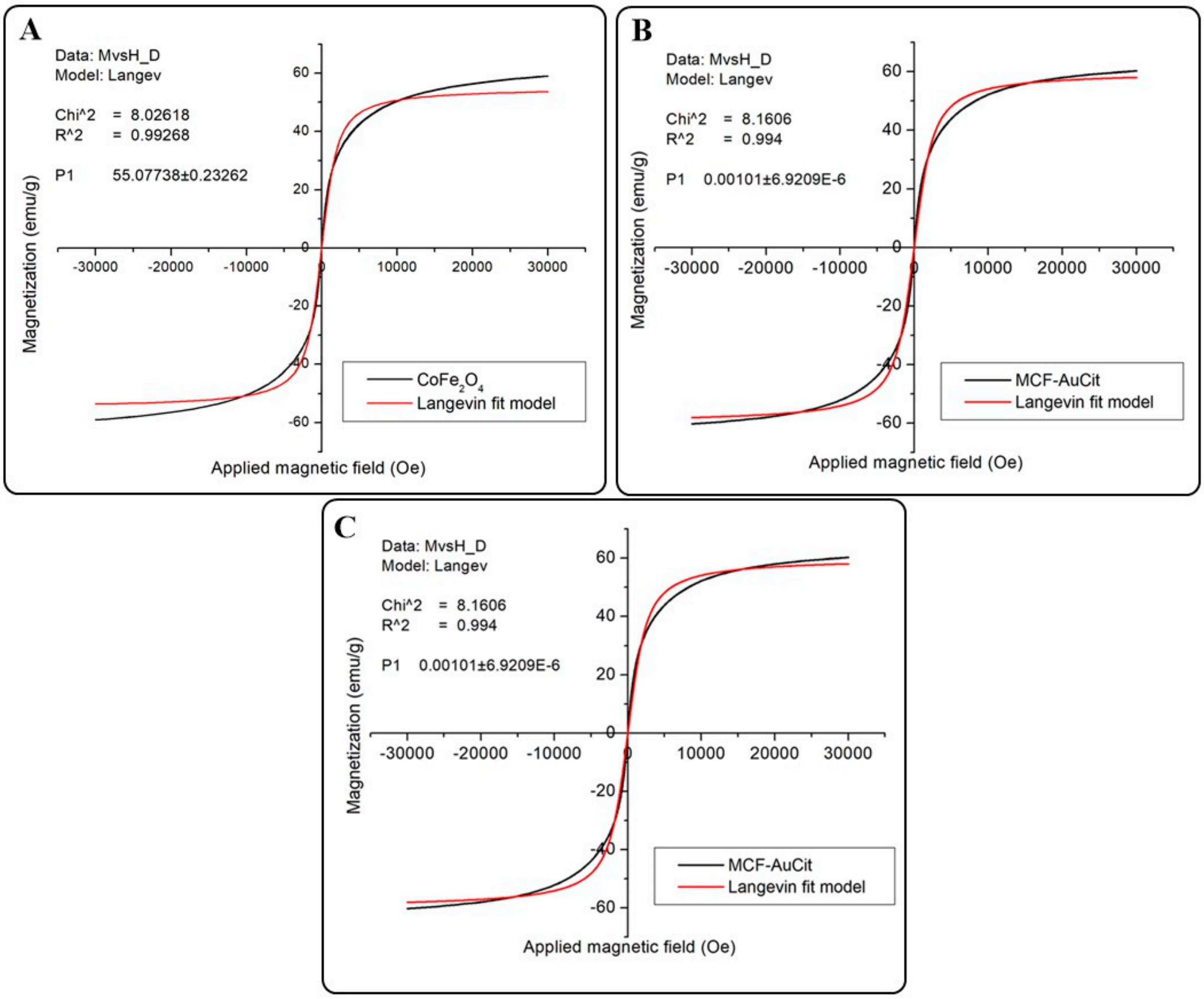

Figure S6. Langevin fit model curve calculated and MCF hysteresis curve.

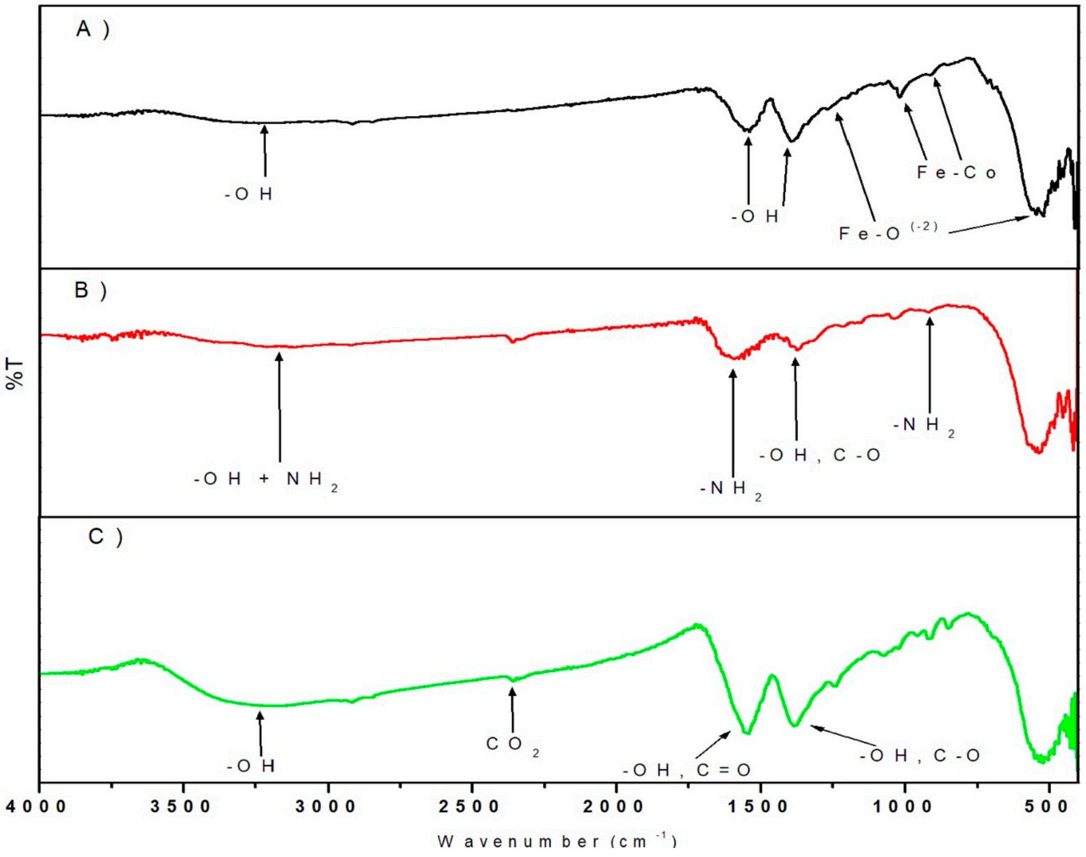

Figure S7. FTIR spectra of a) naked MCF b) MCF-Gly c) MCF-Cit.

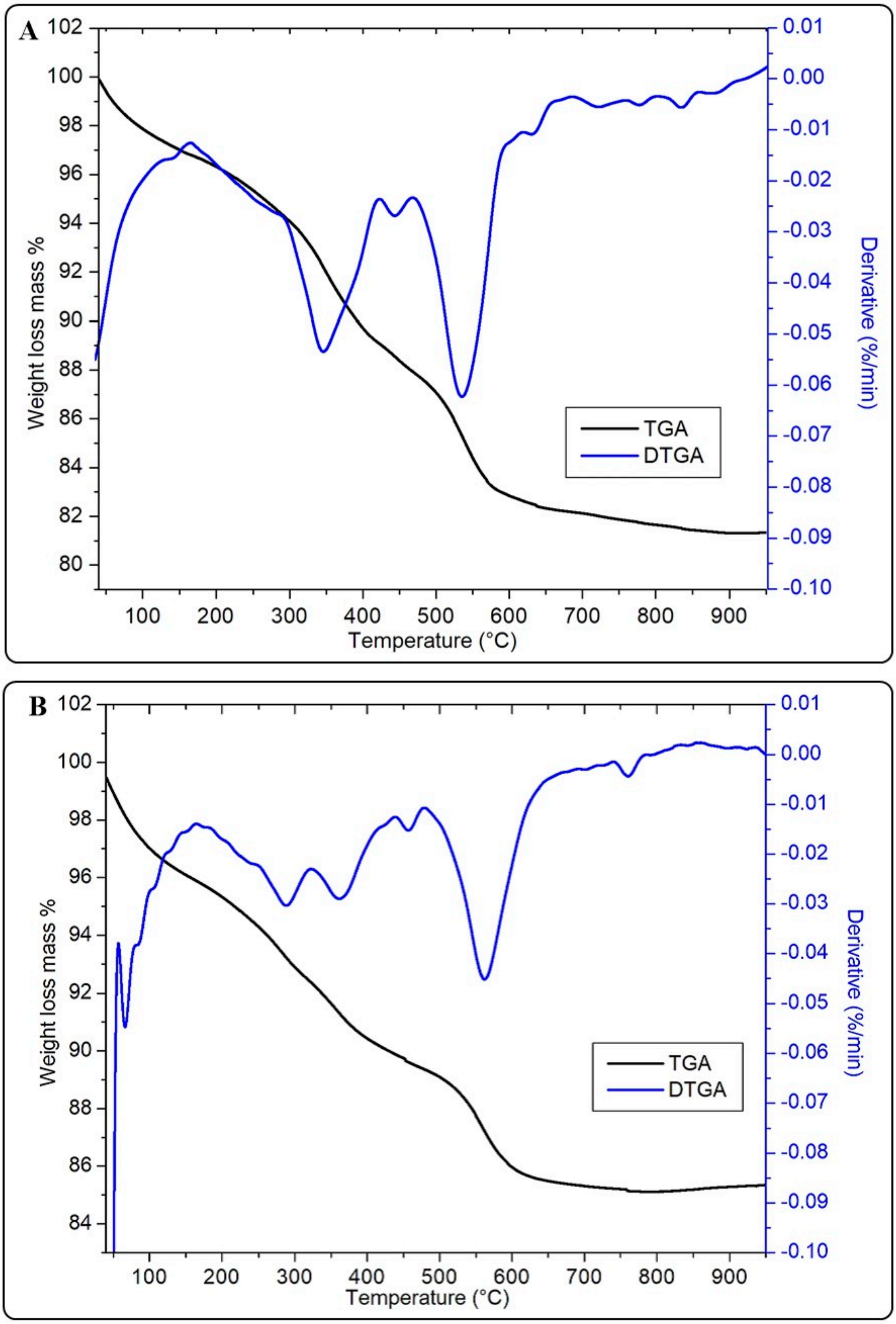
A
B
Weight loss mass %
Derivative (%/min)
Temperature (°C)
TGA
DTGA

Figure S8.TGA and DTGA analyses of **A)** MCF-AuCit and **B)** MCF-AuGly

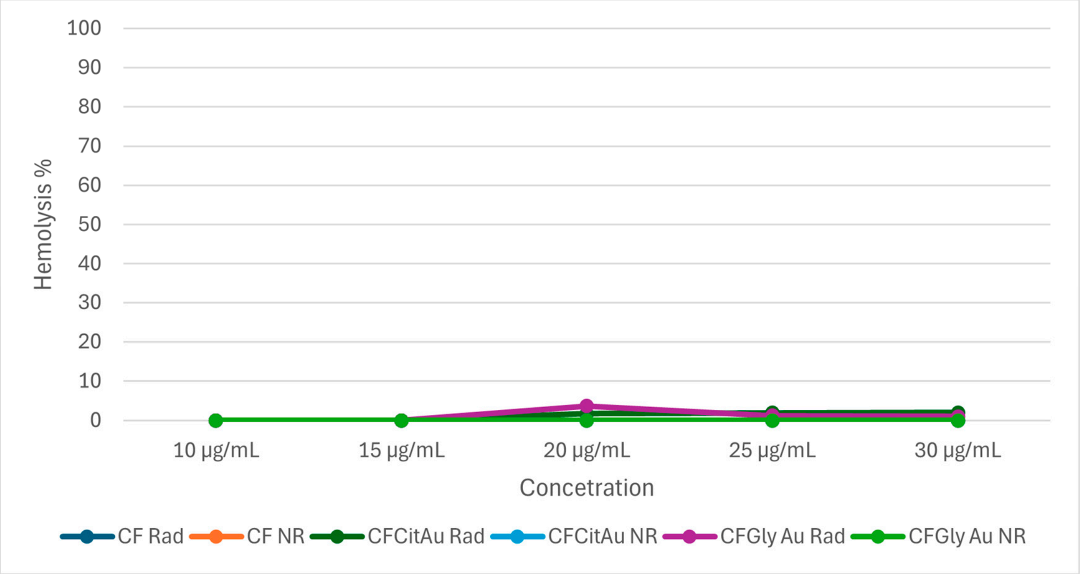

Figure S9. Hemolytic activity results at different concentrations and exposure conditions (1. No-radiated assay; 2. Radiated assay. The groups exposed to MCF-Au were irradiated 10 min with the IR light (850 nm)).

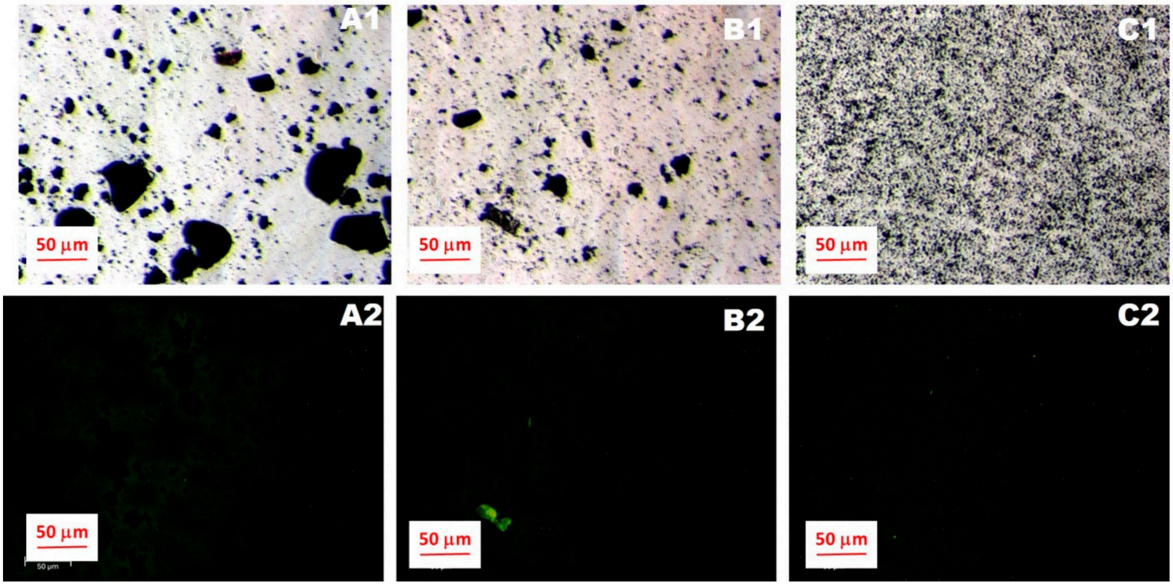

Figure S10: Fluoresence Microscopy images of MCF, MC-AuGly, MCF-Au-Cit. A1, B1, B2: Bright field images of the NMs. A2, B2, C2) Fluorescence images of the NMs after being irradiated with for 10 minutes with IR light.

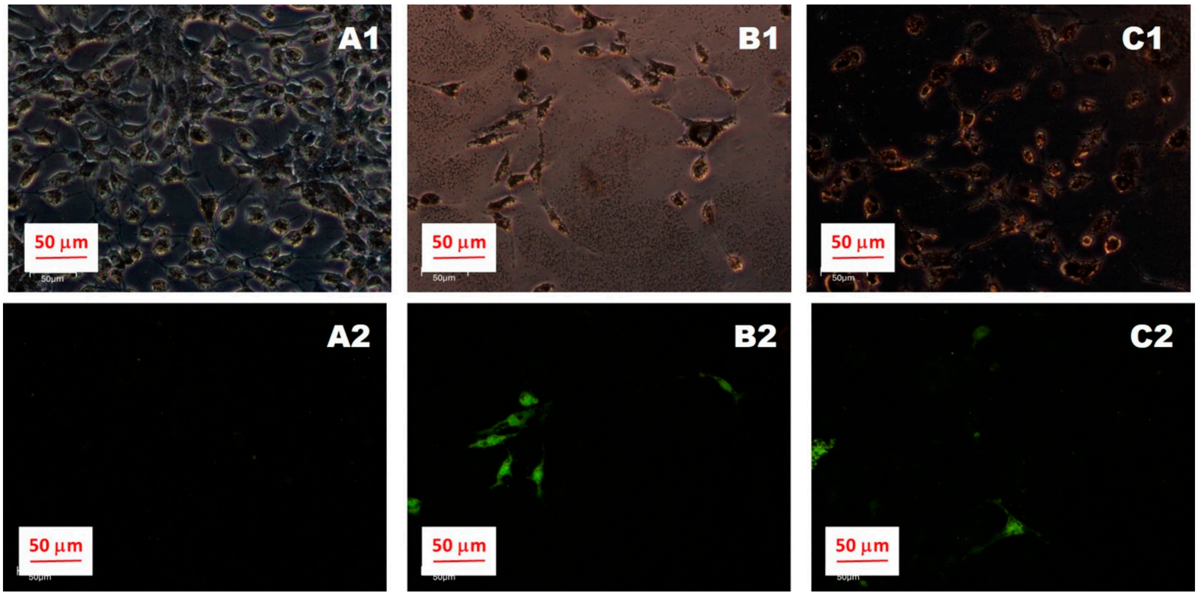

Figure S11: Fluorescence Microscopy images of Detroit 548 cells exposed to MCF-AuGly and radiated with IR light at different time intervals. A1, B1, B2: Bright field images of the Detroit cells (1, 5 and 10 minutes respectively). A2, B2, C2) Fluorescence images of the cells exposed to NMs after being irradiated with for 1, 5 or 10 minutes with IR light (respectively).

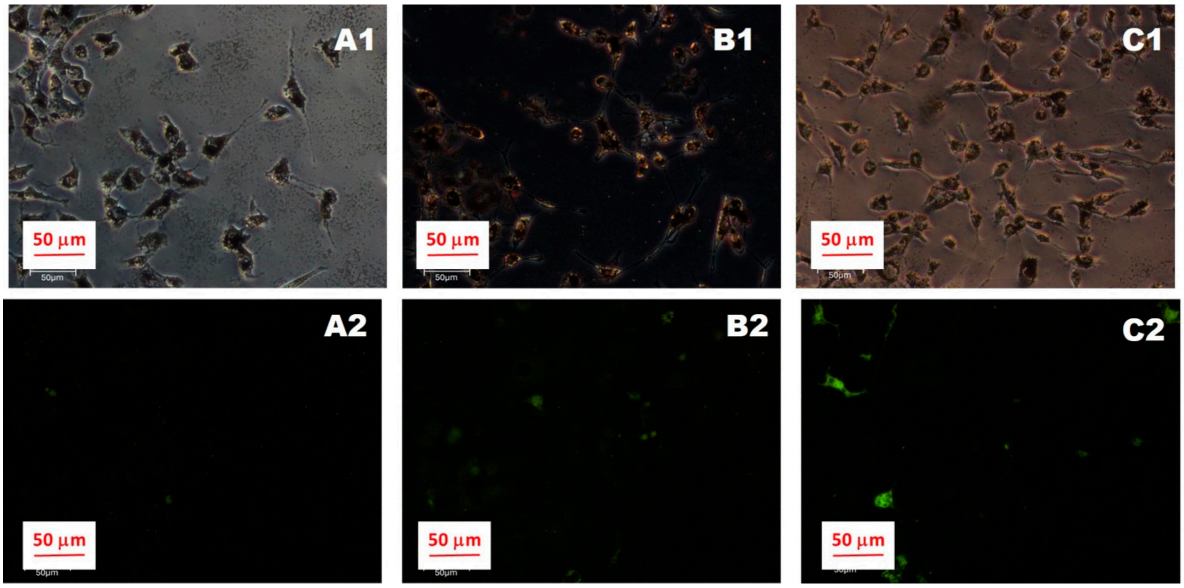

Figure S12: Fluorescence Microscopy images of Detroit 548 cells exposed to MCF-AuCit and radiated with IR light at different time intervals. A1, B1, B2: Bright field images of the Detroit cells (1, 5 and 10 minutes respectively). A2, B2, C2) Fluorescence images of the cells exposed to NMs after being irradiated with for 1, 5 or 10 minutes with IR light (respectively).

.

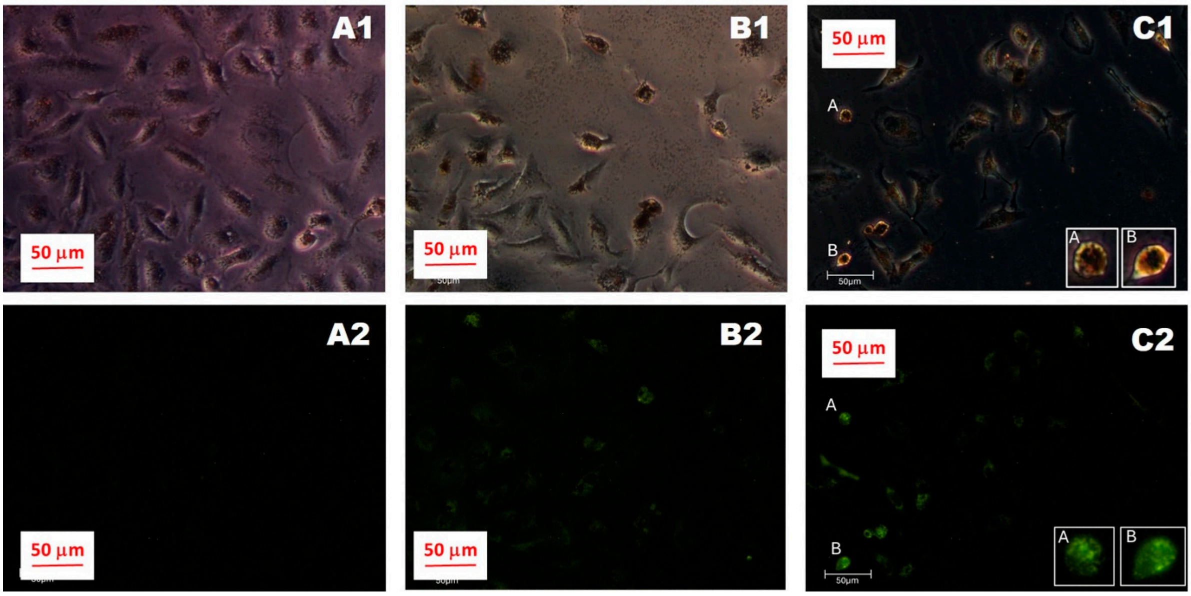

Figure S13: Fluorescence Microscopy images of A549 cells exposed to MC-AuGly and ra- diated with IR light at different time intervals. **A1, B1, B2**: Bright field images of the A549 cells (1, 5 and 10 minutes

respectively). **A2, B2, C2**) Fluorescence images of the exposed cells after being irradiated with for 1, 5 or 10 minutes with IR light (respectively).

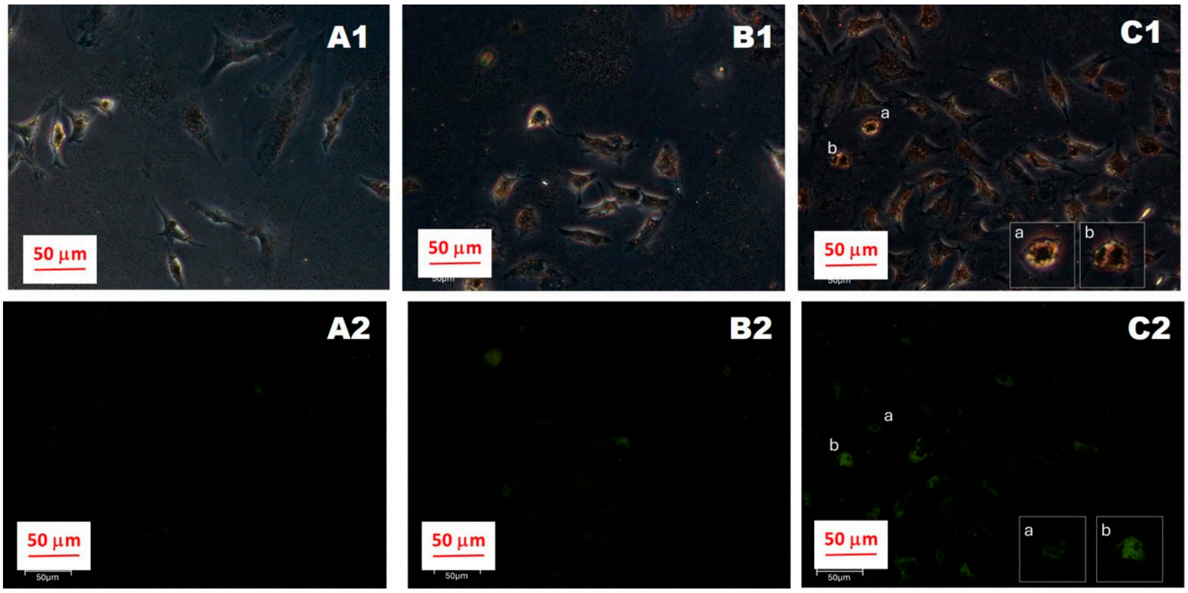

Figure S14: Fluorescence Microscopy images of A549 cells exposed to MC-AuCit and ra- diated with IR light at different time intervals. **A1, B1, B2:** Bright field images of the A549 cells (1, 5 and 10 minutes respectively). **A2, B2, C2**) Fluorescence images of the exposed cells after being irradiated with for 1, 5 or 10 minutes with IR light (respectively).

Table S1. EDX results with their mean values and standard deviation

MCF-Au	Co	Fe	O	Au
**MCF-AuCit**	19±6	44±15	28±21	9±13
**MCF-AuGly**	9±1	22±0	68±1	0.2

Table S2. CCK-8 test of viability results for both cell lines. Rad: IR Light irradiated NR: No irradiated

Sample	0 µg/mL	10µg/mL	15µg/mL	20µg/mL	25µg/mL	30µg/mL
**A549 Cit Rad**	100	**32**	16	13	10	6
**A549 Cit NR**	100	55	**49**	43	36	27

**A549 Gly Rad**	100	71	64	61	51	**45**
**A549 Gly NR**	100	98	91	88	78	72
**D548 Cit Rad**	100	64	58	53	**45**	36
**D548 Cit NR**	100	99	93	88	80	71
**D548 Gly Rad**	100	79	71	69	58	52
**D548 Gly NR**	100	87	79	77	66	61
